# CFTR modulators exert subset-specific phenotype remodeling on circulating neutrophils in cystic fibrosis

**DOI:** 10.1093/immhor/vlag030

**Published:** 2026-07-31

**Authors:** François Chable de la Héronnière, Théo Dhôte, Rodrigo de Oliveira Formiga, Lucile Regard, Giovanni Saraceni-Tasso, Muriel Andrieu, Souganya Many, Jennifer Da Silva, Frédéric Pène, Clémence Martin, Maha Zohra Ladjemi, Pierre-Régis Burgel, Véronique Witko-Sarsat

**Affiliations:** Inserm U1016, Institut Cochin, Paris, France; Université Paris-Cité, Paris, France; Inserm U1016, Institut Cochin, Paris, France; Université Paris-Cité, Paris, France; Service de Pneumologie & Centre de Référence Maladies Rares Mucoviscidose, site coordonnateur, Hôpital Cochin, Assistance Publique-Hôpitaux de Paris-Centre & Université Paris-Cité, Paris, France; ERN-Lung, Cystic Fibrosis Core Network, Frankfurt, Germany; Inserm U1016, Institut Cochin, Paris, France; Université Paris-Cité, Paris, France; Inserm U1016, Institut Cochin, Paris, France; Université Paris-Cité, Paris, France; Service de Pneumologie & Centre de Référence Maladies Rares Mucoviscidose, site coordonnateur, Hôpital Cochin, Assistance Publique-Hôpitaux de Paris-Centre & Université Paris-Cité, Paris, France; ERN-Lung, Cystic Fibrosis Core Network, Frankfurt, Germany; Inserm U1016, Institut Cochin, Paris, France; Université Paris-Cité, Paris, France; Inserm U1016, Institut Cochin, Paris, France; Université Paris-Cité, Paris, France; Inserm U1016, Institut Cochin, Paris, France; Université Paris-Cité, Paris, France; Inserm U1016, Institut Cochin, Paris, France; Université Paris-Cité, Paris, France; Service de Pneumologie & Centre de Référence Maladies Rares Mucoviscidose, site coordonnateur, Hôpital Cochin, Assistance Publique-Hôpitaux de Paris-Centre & Université Paris-Cité, Paris, France; ERN-Lung, Cystic Fibrosis Core Network, Frankfurt, Germany; Inserm U1016, Institut Cochin, Paris, France; Université Paris-Cité, Paris, France; Service de Médecine intensive & Réanimation, Hôpital Cochin, Assistance Publique-Hôpitaux de Paris-Centre & Université Paris-Cité, Paris, France; Inserm U1016, Institut Cochin, Paris, France; Université Paris-Cité, Paris, France; Service de Pneumologie & Centre de Référence Maladies Rares Mucoviscidose, site coordonnateur, Hôpital Cochin, Assistance Publique-Hôpitaux de Paris-Centre & Université Paris-Cité, Paris, France; ERN-Lung, Cystic Fibrosis Core Network, Frankfurt, Germany; Inserm U1016, Institut Cochin, Paris, France; Université Paris-Cité, Paris, France; Inserm U1016, Institut Cochin, Paris, France; Université Paris-Cité, Paris, France; Service de Pneumologie & Centre de Référence Maladies Rares Mucoviscidose, site coordonnateur, Hôpital Cochin, Assistance Publique-Hôpitaux de Paris-Centre & Université Paris-Cité, Paris, France; ERN-Lung, Cystic Fibrosis Core Network, Frankfurt, Germany; Inserm U1016, Institut Cochin, Paris, France; Université Paris-Cité, Paris, France

**Keywords:** CFTR modulator, cystic fibrosis, neutrophils, spectral flow cytometry, neutrophil subsets

## Abstract

A key aspect of cystic fibrosis pathophysiology is the significant role played by neutrophils, central to the inflammatory response in cystic fibrosis airways. Neutrophil-dominated inflammation greatly influences clinical outcomes, including airway damage and lung function decline. People with cystic fibrosis have an increase in circulating neutrophils that exhibit numerous functional and phenotypic abnormalities.

We used spectral flow cytometry to delineate the expression of 24 cell surface markers on neutrophils from 24 healthy donors and 45 adults with cystic fibrosis, before and after elexacaftor-tezacaftor-ivacaftor treatment. This comprehensive analysis examined phenotypic markers associated with fundamental neutrophil functions, including differentiation, maturity, activation, antimicrobial activities (degranulation, pathogen sensing and chemotaxis), metabolism, and immunomodulation.

Prior to treatment, neutrophils from adults with cystic fibrosis displayed a phenotype suggestive of immune activation and metabolic aberration. Treatment with elexacaftor-tezacaftor-ivacaftor normalized a FPR1^high^/CXCR2^high^ subset, an observation consistent with enhanced pathogen-sensing capacity. The treatment, however, failed to restore fully normal phenotypes in cystic fibrosis neutrophils but rather induced the modulation of a PD-L1^high^/CD114^high^/GLUT-1^high^ subset of mature neutrophils with an increased expression of PD-L1 and adhesion molecules such as CD11c and CD11b.

Taken together, our data reveal a specific immunophenotypic signature of neutrophils in cystic fibrosis that is further modified by treatment with elexacaftor-tezacaftor-ivacaftor, suggesting the opportunity for developing adjunctive therapies to enhance beneficial antimicrobial subsets while simultaneously mitigating immunosuppressive phenotypes, thereby opening new perspectives for immune modulation in cystic fibrosis treatment.

## Introduction

Cystic fibrosis (CF) is an autosomal recessive disease caused by loss-of-function variants in the gene encoding the cystic fibrosis transmembrane conductance regulator (CFTR) protein. Dysfunction of the CFTR ion channel leads to the production of thick mucus in the respiratory tract, thereby impairing mucociliary clearance and resulting in mucus plugging and bronchiectasis.^1^ People with CF (pwCF) experience recurrent pulmonary infections with pathogens such as *Pseudomonas aeruginosa* and *Staphylococcus aureus*, triggering harmful inflammation and frequent acute pulmonary exacerbations that accelerate lung function decline and can ultimately lead to respiratory failure.

A key component of CF pathophysiology is the deleterious roles that neutrophils play in their inherent defective antibacterial action and in their proinflammatory activities, which together amplify airway inflammation.^2^^,^^3^ Neutrophil-dominated inflammation drives progression of CF, by promoting airway damage and accelerating decline in lung function. Human neutrophils express the CFTR protein, which supports the generation of myeloperoxidase (MPO)-dependent oxidants by transporting chloride into the phagosome and thereby enabling production of the potent microbicidal, hypochlorous acid (HOCl).^4^^,^^5^ The chronic bacterial airway infections in pwCF reflect in part the lack of HOCl production.^3^ In addition to its direct role in undermining antibacterial defenses, CFTR dysfunction compromises multiple neutrophil processes, resulting in altered degranulation and phagosomal pH,^6^ and excessive production of neutrophil extracellular traps (NETs).^7^ Moreover, delayed physiological apoptosis of CF neutrophils^8^ prolongs their survival in tissues and thus delays the timely resolution of inflammation.^9^

One of the most significant changes in our understanding of neutrophil biology is the discovery of the functional heterogeneity of neutrophils, which contrasts with the longstanding view of these phagocytes as a homogeneous population with uniform phenotype and functional potential.^10^ Several comprehensive immunophenotypic and transcriptional analyses, both at steady state and upon stress, have identified heterogeneity among circulating neutrophils with respect to phenotypic and functional state.^11^ This extreme plasticity in vivo includes the presence of specific immunosuppressive subsets in circulating neutrophils from patients with autoimmune or inflammatory diseases, with cancer or in G-CSF-treated donors.^12^ We previously employed spectral flow cytometry to characterize the diversity of immunophenotypes of circulating CF neutrophils at steady state. We identified specific subsets of activated and immature neutrophils, an increased immunosuppressive CD16^high^/CD62L^low^ subset, and a unique PD-L1/CD114 subpopulation in pwCF.^13^ In addition, we observed that these immune alterations are differentially modulated during CF exacerbations, although a mechanistic understanding of neutrophil diversity in CF is still lacking.

The recent introduction of the combination of CFTR modulators elexacaftor-tezacaftor-ivacaftor (ETI) represents a significant therapeutic breakthrough in CF. It has shown promising results in improving not only respiratory function but also systemic inflammation.^14^ This treatment enhances the trafficking of mutated CFTR to the cell membrane and partially restores its function. However, ongoing lung infections and inflammation remain major challenges.^15^^,^^16^ Furthermore, key neutrophil activities, such as production of reactive oxygen species (ROS), chemotaxis, and phagocytosis, appear largely unchanged by ETI,^17^ and the transcriptomes of circulating CF neutrophils are persistently primed despite ETI treatment.^18^ Nonetheless, ETI treatment significantly decreases interleukin-8 and neutrophil elastase levels in sputum from pwCF^19^ and normalizes the number of circulating neutrophils in adults with advanced CF.^20^

A critical yet unresolved question in CF research is how ETI influences neutrophil dysfunction and whether its effects vary across different neutrophil subsets. Addressing this gap in knowledge is essential for optimizing anti-inflammatory strategies for the care of pwCF.^21^ Our findings offer novel insights into CF-associated neutrophil plasticity before and after ETI with potential functional implications.

## Subjects and methods

### Study participants

Forty-five adults (≥18 yr) including 22 males (48.8%) and 23 females (51.2%) with CF were recruited in clinically stable condition from the French National CF Reference Centre at Cochin Hospital (Paris, France). This study was approved by the Ethics committee (Committee for the Protection of Persons no. 19005 –2137-18.11.26. 50423). Each participant provided written informed consent. The inclusion period spanned from July 08, 2021, to May 24, 2022. The main characteristics of the CF population at the time of ETI initiation are described in Table 1. A first visit (V1) occurred within days prior to ETI initiation, and a second visit (V2) was performed after 1 to 12 mo with ETI (with a mean of 86 ± 52.1 d). As expected, initiation of ETI resulted in reduced sweat chloride concentration, improved the lung function (ppFEV_1_) and increased body mass index; these effects occurred similarly in males and in females (Fig. S1A, B, and C). Twenty-four healthy donors (HD) including 11 males (45.8%) and 13 females (54.2%) were recruited from the French blood bank (Etablissement Français du Sang) and were significantly older than pwCF with a mean age ± SEM of 44.6 ± 3.3 (*n* = 24) versus 35.4 ± 1.7 (*n* = 45), respectively (*P* < 0.01, Student *t* test).

**Table 1:** Demographic and clinical characteristics of pwCF.

Adults patients with cystic fibrosis (*n* = 45)	
**Age in years, mean (SD)**	35.4 (11.3)
**Sex at birth, number of males (%)**	22 (48.8)
**Weight in kg at V1, mean (SD)**	68.2 (16.4)
**Weight in kg at V2, mean (SD)**	68.8 (14.5)
**CFTR genotype –F508del copies number**	
**2 copies**	11 (24.4)
**1 copy**	34 (75.6)
**Sweat chloride concentration (expressed in mMol/l) mean (SD)**	90.7 (24.0)
**Respiratory scores**	
**ppFEV_1_ % at V1, mean (SD)**	71.4 (22.9)
**ppFEV_1_ % at V2, mean (SD)**	84.2 (22.5)
**Bronchial bacterial colonization Sputum bacteria, n (%)**	
***P. aeruginosa***	16 (35.6)
***M. abscessus***	2 (4.4)
**Allergic bronchopulmonary aspergillosis**	16 (35.6)
**Medication, *n* (%)**	
**Azithromycin**	21 (46.7)
**Oral corticosteroid**	2 (4.4)
**Anti-IL5 or anti-IL5-R Ab**	7 (15.6)
**CFTR modulators prior to ETI, *n* (%)**	15 (33.3)
**Ivacaftor**	1 (2.2)
**Lumacator-Ivacaftor**	5 (11.1)
**Tezacaftor-Ivacaftor**	9 (20)
**Antibiotic course in the last year, mean (SD)**	0.93 (1.65)
**Antibiotic days in the last year, mean (SD)**	12.3 (20.7)
**Time interval between V1 and V2, days, mean (SD)**	86.6 (52.1)

Additional information on clinical parameters and effects of ETI according to the sex at birth of pwCF is shown in Fig. S1. Abbreviations: V1, Visit 1 before initiation of ETI treatment; V2, Visit 2 after ETI treatment; *n*, number of individuals; SD, standard deviation.

### Measurement of clinical parameters

Sweat chloride concentration from pwCF was measured using pilocarpine iontophoresis according to established clinical guidelines. Sweat was stimulated by pilocarpine application with a mild electric current, collected over a 30-min period, and chloride concentration was determined using chloridometer titration.^22^^,^^23^

Pulmonary function of pwCF was assessed by spirometry according to American Thoracic Society/European Respiratory Society (ATS/ERS) standards. Forced expiratory volume in 1 s (FEV_1_) was expressed as percent predicted (ppFEV_1_) using Global Lung Initiative (GLI) reference equations adjusted for age, sex, height, and ethnicity.^24^^,^^25^

### Neutrophil immune-labeling and flow cytometry analysis

Peripheral blood was collected into Vacutainer EDTA K2 (1.8 mg/mL, BD Biosciences, Franklin Lakes, New Jersey, USA) from HD and pwCF before (V1) and after (V2) the initiation of ETI treatment. The phenotypic characterization of neutrophil subpopulations was performed within whole blood to avoid any artefactual activation. This analysis was carried out by multicolor flow cytometry using a spectral flow cytometer (5-laser configuration Aurora, Cytek Biosciences, Fremont, California, USA) allowing in-depth immunophenotyping of 24 surface markers (Table S1). Samples were processed within 4 h of collection. First, the antibody mix was prepared, with one tube containing 24 specific antibodies. The antibody was added to the cells and incubated for 15 min on ice in the dark. Red blood cells were then lysed with lysing buffer 1X (BD Biosciences, 555899) and washed with PBS. To fix the cells, 200 µL of 1% paraformaldehyde was added to each tube. Finally, 300 µL of stain buffer (BD Biosciences) was added for storage at +4 °C until acquisition within 24 h of labeling. During spectral unmixing, cell autofluorescence was explicitly extracted using appropriate unstained controls at the individual level. Therefore, MFI values represent relative autofluorescence-corrected intensities. Instrument performance was monitored daily using standard quality control procedures (using Cytek QC beads) ensuring minimal signal drift across runs and stable performance throughout the study. Granulocytes were first selected by size (FSC) and granularity (SSC). After exclusion of doublets (SSC-H/SSC-A and FSC-H/FSC-A), CD15 positive cells resulted in the neutrophil population. Finally, residual eosinophils were excluded using their higher Siglec 8 expression (Fig. S2A). The neutrophil specificity of gating strategy was verified by backgating analysis using size/granularity and CD66b/CD14/CD15 expressions to allow visualization of granulocytic, monocytic and lymphoid populations (Fig. S3).

### Analysis using visual interactive t-distributed stochastic neighbor embedding (Vi-tSNE)

In order to identify and to visualize neutrophil subsets, an unsupervised analysis was performed using the dimensional reduction algorithm Visual interactive t-Distributed Stochastic Neighbor Embedding (Vi-tSNE) to define clusters of markers characteristic of specific neutrophil subsets as described in Fig. S2B. This analysis was carried out using the CytoBank platform (Beckman Coulter Life Sciences, Brea, California, USA). A sample of events was randomly taken from each pwCF (HD: 6,101/individual, V1: 5,262/individual, V2: 7,132/individual). All events were then grouped into concatenated files (HD: 140,323 events, V1: 236,745 events, V2: 313,802 events). A total of 140,000 random events for each group were then subjected to the algorithm to form clusters according to the expression levels of the surface phenotyping markers, using a rainbow heat scale to represent expression intensity, from red indicating high expression levels to blue indicating low expression levels. For every individual (either HD or pwCF), cluster identity was systematically verified by backgating and the individual enrichment of clusters among total neutrophils was reported.

### Statistics

Statistical analyses and graphs were performed with Prism version 10 (GraphPad Software, San Diego, California, USA). To compare HD with pwCF at V1 and at V2, non-parametric Mann–Whitney tests were used when data distribution was not normal, while parametric unpaired *t* tests were used for data presenting a normal distribution. To compare V1 with V2, non-parametric Wilcoxon tests were used when data distribution was not normal and with less than 30 individuals, while paired *t* tests were used for data with more than 30 individuals and presenting a normal distribution. The *P* values were corrected for multiple comparison using the false discovery rate (FDR) method. Differences were considered statistically significant when *P* < 0.05. Correlation coefficients were calculated using linear regression and the associated *P* values were obtained using the Student *t* test.

## Results

### Baseline phenotype of CF neutrophils

**Figure 1:**
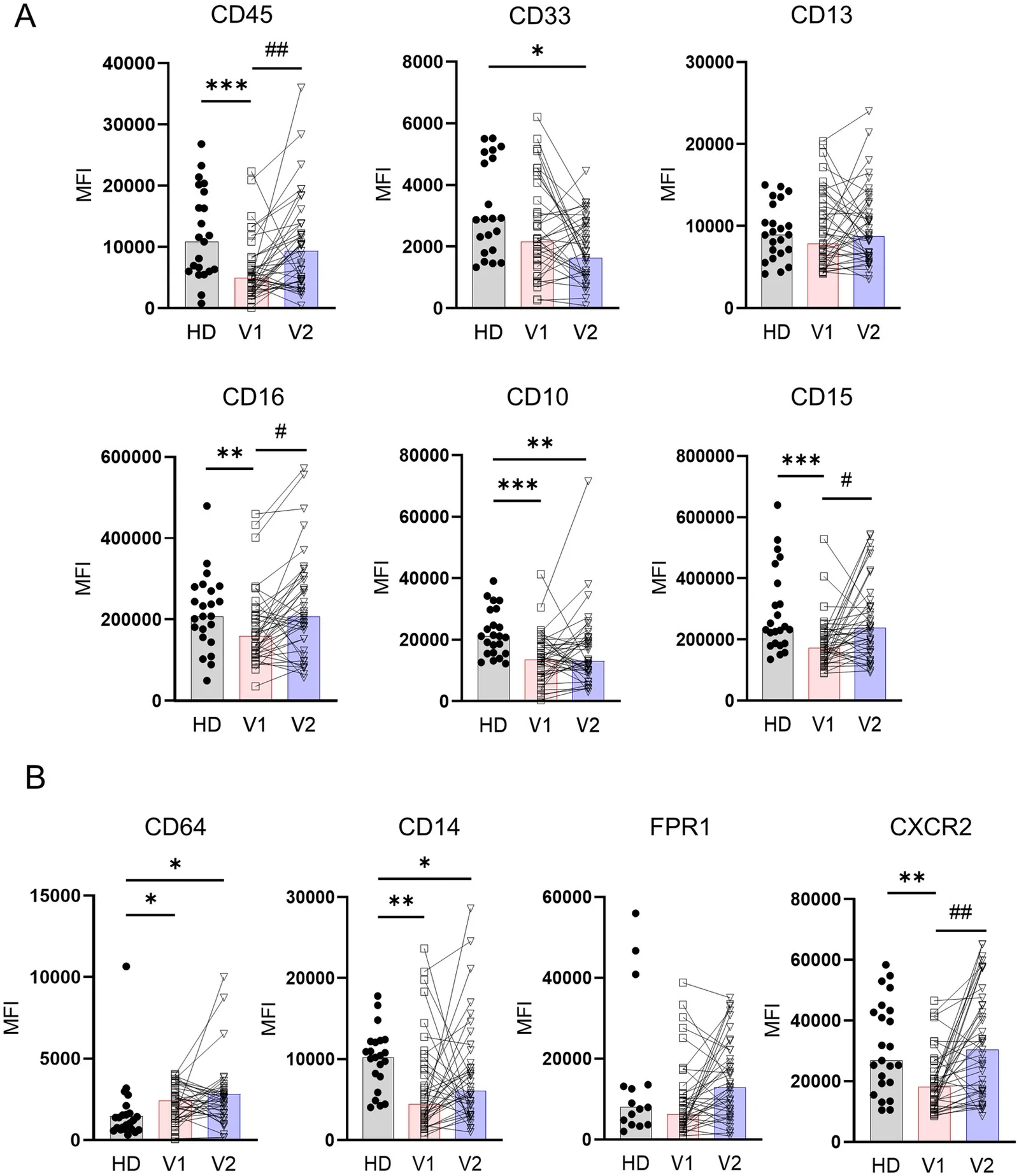
Analysis of the expression of surface markers associated with differentiation and pathogen sensing in neutrophils from HD and from pwCF before (V1) and after (V2) ETI treatment. Analysis of surface marker expressions related to (A) differentiation (CD45, CD33, CD13) and maturity (CD16, CD10, CD15) and (B) innate pathogen sensing (CD64, CD14, FPR1) and chemotaxis (CXCR2) was performed on whole blood circulating neutrophils from HD and from pwCF at V1 and at V2. Data are Mean Fluorescence Intensities (MFI) measured for each subject and group medians are represented with bars. Statistical analysis has been performed either using unpaired Mann-Whitney test when comparing HD vs V1 and HD vs V2 (*P < 0.05, **P < 0.01, ***P < 0.001) or using paired Wilcoxon test when comparing V1 vs V2 (#*P* < 0.05, ##*P* < 0.01). The *P* values were corrected for multiple comparison using the false discovery rate (FDR) method.

**Figure 2:**
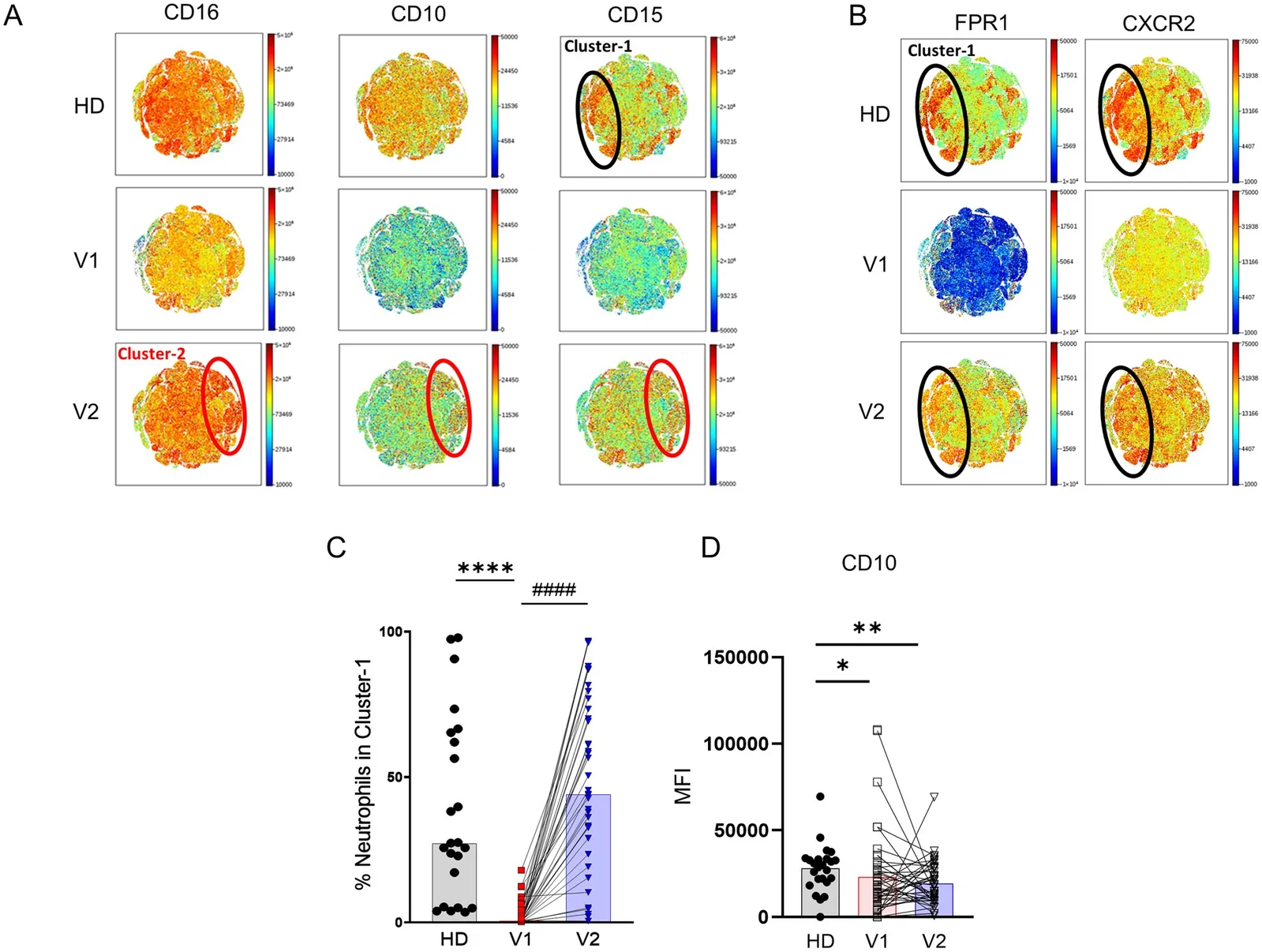
Unsupervised analysis to identify neutrophil subpopulations based on maturity, differentiation and antibacterial response markers and effect of ETI treatment. Visualization of expression clusters in neutrophils from HD and pwCF at V1 and V2 using Visual interactive t-Distributed Stochastic Neighbor Embedding (Vi-tSNE) for (A) CD16, CD10 and CD15 and (B) FPR1 and CXCR2. Plots are represented following a rainbow heat scale for fluorescence intensity with the red for the highest and the blue the lowest value of intensity. Cluster-1 and Cluster-2 are indicated with black and red circles, respectively. (C) Percentages of FPR1^high^/CXCR2^high^ in neutrophils from Cluster-1 in HD and in pwCF at V1 and V2. Individual data are shown for each subject, and group medians are represented with bars. (D) Analysis of CD10 expression in neutrophils from Cluster-1. Data are Mean Fluorescence Intensities (MFI) measured for each subject and group medians are represented with bars. Statistical analysis has been performed either using unpaired Mann–Whitney test when comparing HD vs V1 and HD vs V2 (*P < 0.05, **P < 0.01, ****P < 0.0001) or using paired Wilcoxon test when comparing V1 vs V2 (####*P* < 0.0001).

**Figure 3:**
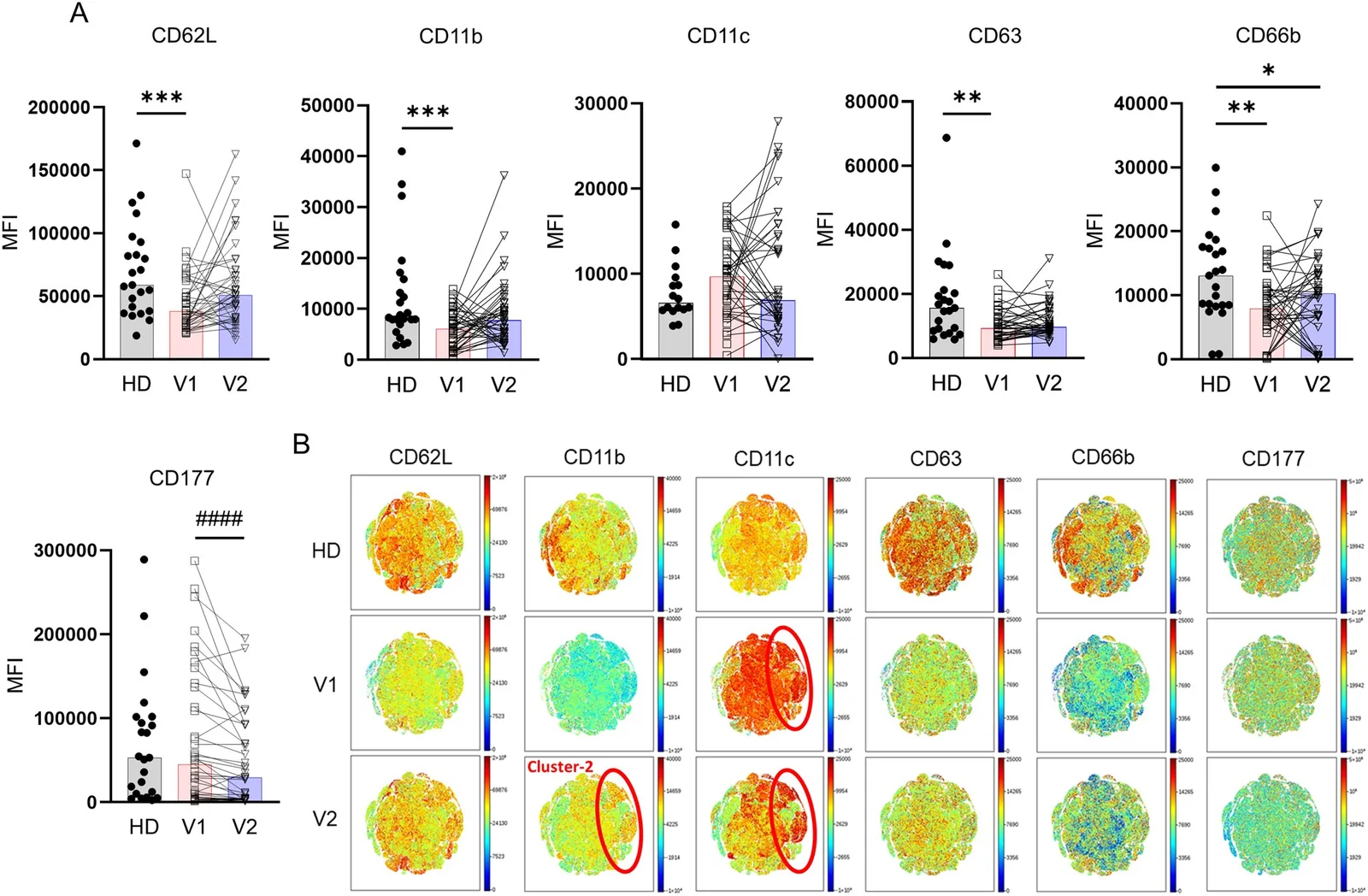
Analysis of the expression of surface markers and related clusters associated with activation and degranulation in neutrophils before (V1) and after (V2) ETI treatment. (A) Analysis of individual surface marker expressions related to activation (CD62L, CD177), adhesion (CD11b, CD11c) and degranulation (CD63, CD66b) was performed on whole blood circulating neutrophils from HD and from pwCF at V1 and V2. Data are Mean Fluorescence Intensities (MFI) measured for each subject and group medians are represented with bars. Statistical analysis has been performed either using unpaired Mann-Whitney test when comparing HD vs V1 and HD vs V2 (*P < 0.05, **P < 0.01, ***P < 0.001) or using paired Wilcoxon test when comparing V1 vs V2 (####*P* < 0.0001). The *P*-values were corrected for multiple comparison using the false discovery rate (FDR) method. (B) Visualization of expression clusters in neutrophils from HD and from pwCF at V1 and V2, using Visual interactive t-Distributed Stochastic Neighbor Embedding (Vi-tSNE) for CD62L, CD11b, CD11c, CD63, CD66b, and CD177. Plots are represented following a rainbow heat scale for fluorescence intensity with the red for the highest and the blue the lowest value of intensity. Cluster-2 is indicated with a red circle.

**Figure 4:**
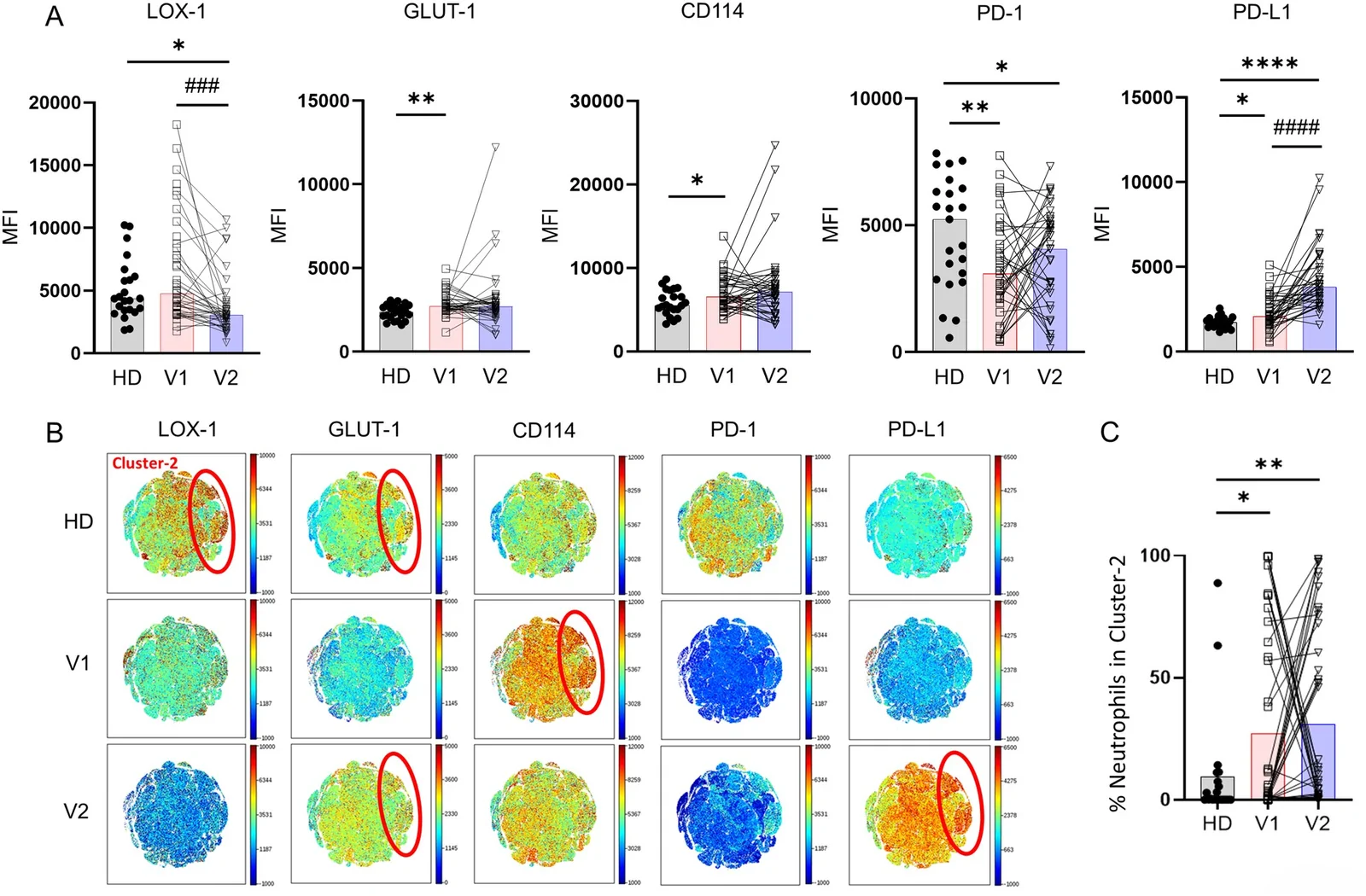
Analysis of the expression of metabolic and immunomodulatory markers with their associated clusters in neutrophils before (V1) and after (V2) ETI treatment. (A) Analysis of surface marker expressions related to metabolism (LOX-1, GLUT-1, CD114) and immunosuppression (PD-1, PD-L1) was performed on whole blood circulating neutrophils from HD and from pwCF at V1 and V2. Data are Mean Fluorescence Intensities (MFI) measured for each subject and group medians are represented with bars. Statistical analysis has been performed either using unpaired Mann–Whitney test when comparing HD vs V1 and HD vs V2 (*P < 0.05, **P < 0.01, ***P < 0.0001) or using paired Wilcoxon test when comparing V1 vs V2 (###*P* < 0.001, ####*P* < 0.0001). The *P* values were corrected for multiple comparison using the false discovery rate (FDR) method. (B) Visualization of expression clusters in neutrophils from HD and from pwCF at V1 and V2, using Visual interactive t-Distributed Stochastic Neighbor Embedding (Vi-tSNE) for LOX-1, GLUT-1, CD114, PD-1 and PD-L1. Plots are represented following a rainbow heat scale for median fluorescence intensity with the red for the highest and the blue the lowest value of intensity. Cluster-2 is indicated with a red circle. (C) Percentages of neutrophils within Cluster-2 among total neutrophils in HD and in pwCF at V1 and V2. Individual data is shown for each subject and group medians are represented with bars. Statistical analysis has been performed either using unpaired Mann–Whitney test when comparing HD vs V1 and HD vs V2 (*P < 0.05) or using paired Wilcoxon test when comparing V1 vs V2 (##*P* < 0.01).

**Figure 5:**
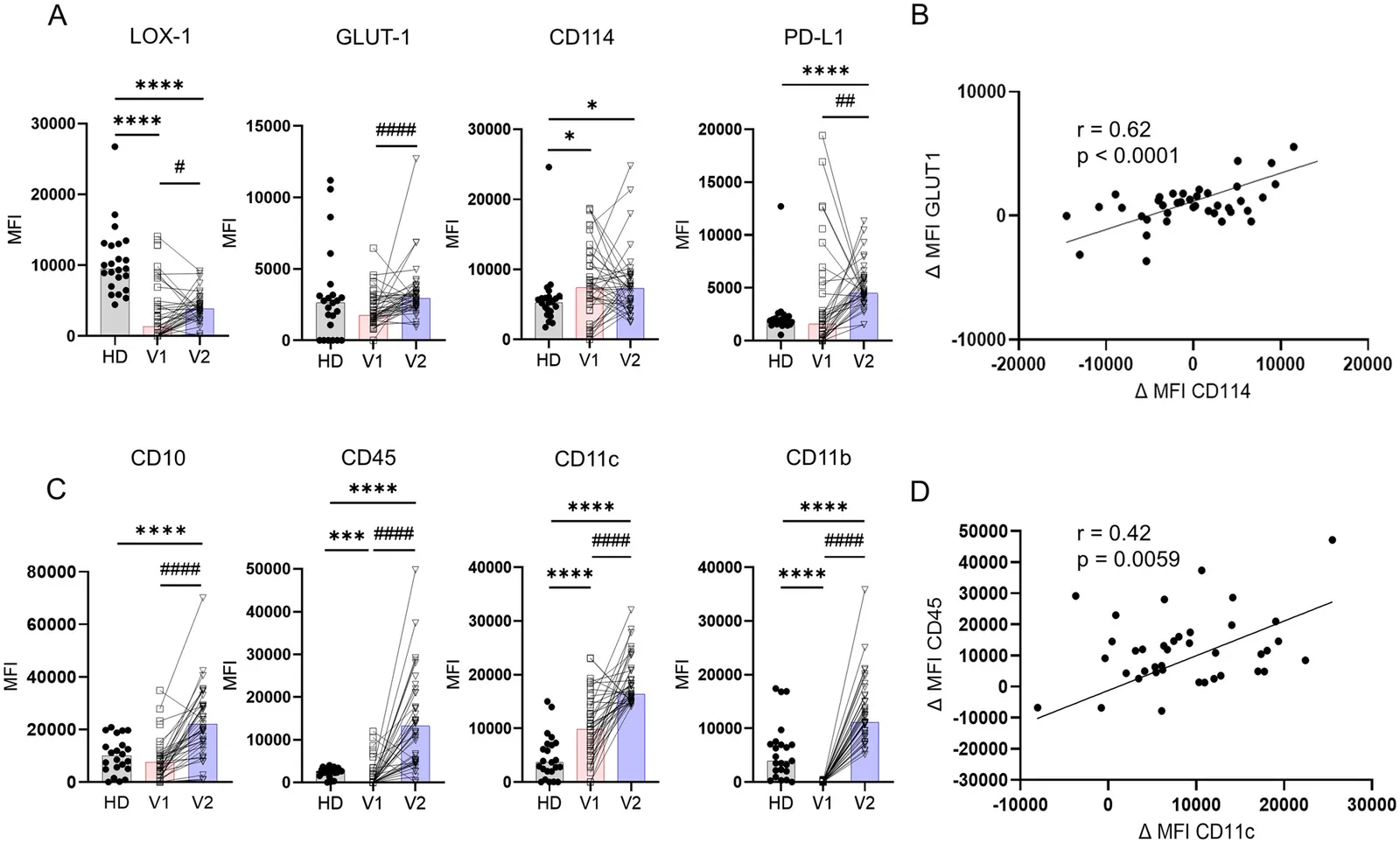
Analysis of the expression of metabolic, immunomodulatory, maturity, and adhesion markers in neutrophils within the Cluster-2 and effect of ETI. (A) Analysis of individual surface marker expressions related to metabolism (LOX-1, GLUT-1, CD114) and to immunosuppression (PD-L1) in the Cluster-2. (B) Correlation between the variation in GLUT-1 and CD114 expressions before and during ETI treatment (V2-V1) in the Cluster-2 in neutrophils from pwCF. (C) Analysis of individual surface marker expressions related to differentiation (CD10 and CD45) and adhesion (CD11b and CD11c) in the Cluster-2. (D) Correlation between the variation in CD45 and CD11c expressions before and during ETI treatment (V2-V1) in the Cluster-2 in neutrophils from pwCF. For (A) and (C) Mean Fluorescence Intensities (MFI) are represented for each marker in the Cluster-2 from HD and from pwCF at V1 and V2. Individual data is shown for each subject and group medians are represented with bars. Statistical analysis has been performed either using unpaired Mann–Whitney test when comparing HD vs V1 and HD vs V2 (*P < 0.05, **P < 0.01, ***P < 0.001, ****P < 0.0001) or using paired Wilcoxon test when comparing V1 vs V2 (#*P* < 0.05, ##*P* < 0.01, ####*P* < 0.0001). The *P* values were corrected for multiple comparison using the false discovery rate (FDR) method. For (B) and (D), correlation coefficients calculated using linear regression and *P*-values using the Student t test are displayed.

By virtue of our study design, we were able to examine a broad profile of phenotypic markers in HD vs untreated pwCF, before (V1) and after initiation of ETI (V2). As detailed in methods, we classified the 24 markers into 5 categories (Table S1), including those describing maturity (Figs. 1A and 2A), pathogen sensing (Figs. 1B, 2B, 2C), activation (including degranulation and adhesion) (Fig. 3), metabolism and immunomodulation (Figs. 4 and 5), and inhibitory receptors (Fig. S4). Compared to HD, neutrophils from pwCF before introduction of ETI expressed either similar marker expression levels (CD33, CD13, FPR1 [Fig. 1A and B], CD11c, CD177 [Fig. 3A], LOX-1 [Fig. 4A]) or lower expression levels (CD45, CD16, CD10, CD15, CD14, CXCR2 [Fig. 1A and B], CD62L, CD11b, CD63, CD66b [Fig. 3A], PD-1 [Fig.4A]), with only five exceptions where there was greater expression on V1 than on HD: one marker in pathogen sensing (CD64) (Fig. 1B), 3 markers in metabolism and immunomodulation (GLUT-1, CD114 and PD-L1) (Fig. 4A) and one inhibitory receptor (Siglec-8) (Fig. S4). Particularly, CD10 had a lower expression in untreated pwCF that was not corrected after ETI, suggesting an increase in immature neutrophils (Fig. 1A). The percentage of CD10 positive cells was also decreased in CF and not corrected after ETI (Fig. S5).

### Effects of ETI on the phenotype of CF neutrophils and restoration of markers to normal levels

In contrast to the predominantly reduced expression of phenotypic markers on neutrophils from pwCF before ETI in comparison to HD neutrophils, the relative expression of phenotypic markers on neutrophils from pwCF after initiation of ETI was far more varied. For example, 3 of the 6 markers for maturity (CD45, CD16, and CD15) were expressed at higher levels in V2 than in V1, whereas CD33 and CD10 expressions were similar in both V2 and V1 (Fig. 1A). In like fashion, the relative expression of markers for pathogen sensing and chemotaxis was greater in V2 than in V1, although this increased expression was statistically significant only for CXCR2 (Fig. 1B). Likewise, the relative expression of markers for degranulation was greater in V2 for CD66b as well as for CD62L and CD11b (although not statistically significant), but lower in V2 for CD177 and nearly the same for CD63 and CD11c (Fig. 3A). Notably, the most significant modulation by ETI was on both LOX-1 and PD-L1, which were expressed at lower and at higher levels in V2 than in V1, respectively (Fig. 4A).

An important intention of our study was to determine if treatment with ETI restores phenotypic marker expressions in neutrophils from pwCF to levels expressed on HD neutrophils. Overall, as judged by assessing individual markers alone and compared to HD, neutrophils from pwCF after introduction of ETI presented restored expression levels of 11 of the 24 assessed markers, including those associated with maturity (CD45, CD16, and CD15) (Fig. 1A), pathogen sensing (FPR1 and CXCR2) (Fig. 1B), degranulation (CD62L, CD11b and CD63) (Fig. 3A), metabolism (GLUT-1 and CD114) (Fig. 4A) and inhibitory receptors (Siglec-8) (Fig. S4). Strikingly, both LOX-1 and PD-1 expressions remained significantly lower on V2 than on HD, whereas PD-L1 expression was increased in V2 compared with HD and with V1 (Fig. 4A).

### Identification of an immature FPR1^high^/CXCR2^high^ neutrophil subset differentially expressed in pwCF and expanded by ETI treatment

To further explore neutrophil heterogeneity, we used unsupervised Vi-tSNE analysis to identify specific clusters of markers that could define neutrophil subsets. In HD, CD15^high^ neutrophils formed a distinct subset with a strong co-expression of the FPR1 and CXCR2 markers (Cluster-1, black-circled, Fig. 2A and B). This FPR1^high^/CXCR2^high^ Cluster-1 enrichment was markedly reduced in V1 and restored in V2 (Fig. 2C). CD10 expression in FPR1^high^/CXCR2^high^ Cluster-1 was decreased in V1 and V2 compared to HD, suggesting that Cluster-1 defined a subset of immature neutrophils and that ETI did not correct the stage of immaturity in this specific cluster (Fig. 2D).

### ETI treatment triggered a maturity and metabolic switched cluster-2 of neutrophils from pwCF with a significant increase of PD-L1 expression

Unsupervised Vi-tSNE analysis was used to examine whether the metabolic and the immunomodulation dysregulation could be more pronounced in a specific neutrophil subset. Accordingly, we identified a distinct “Mickey ears-shape” Cluster-2 (circled in red, Fig. 4B) that delineates a neutrophil subset characterized by a high plasticity in all three groups. This cluster, present in HD, was expanded in pwCF and remained enriched after ETI treatment (Fig. 4C). However, paired individual trajectories revealed substantial inter-individual variability, with some pwCF exhibiting increased whereas others showed decreased Cluster-2 enrichment after ETI treatment. This effect was not associated with age, sex, treatment duration, sweat chloride levels or ppFEV_1_ (data not shown). Most interestingly, Cluster-2 showed a differential switch in metabolic and in immunomodulatory phenotype between HD and CF neutrophils, defined by (i) a significant lower expression of LOX-1 in neutrophils from pwCF in V1 and V2 compared to HD, (ii) a higher expression of CD114 in V1 and V2 and a dramatic increase in PD-L1 expression specifically in V2 compared to HD (Fig. 5A). The variation of expression between V1 and V2 of GLUT-1 expression was correlated with that of CD114, suggesting a coordinated modulation of these metabolic markers (Fig. 5B). Particularly, the expressions of CD45 and CD10 were significantly increased by ETI in Cluster-2 thereby reversing the defect in maturity observed in V1 in neutrophils from pwCF (Fig. 5C). Notably, Cluster-2 in V2 was also characterized by increased expression of CD16 and CD15 markers thereby corroborating that ETI treatment had a cluster-dependent effect on neutrophil maturation (Fig. 2A). Cluster-2 did not overlap with Cluster-1 and did not include neutrophils with a high expression of FPR1 and CXCR2 (Fig. 2B) but showed a selective enrichment of neutrophils with high CD11c expression both in V1 and in V2 (Fig. 3B). Regarding expression of adhesion molecules in Cluster-2 in V1, CD11b expression was significantly decreased compared to HD, whereas CD11c expression was significantly increased (Fig. 5C). In V2, however, a dramatic increase of both CD11c and CD11b expressions, becoming significantly higher compared to V1 and to HD, was observed. The variations between V1 and V2 in CD11c expression correlated positively with those in CD45 expression (Fig. 5D), suggesting a coordinated modification of functions related to the expression of adhesion molecules involved in cell-cell interaction and in maturation.^26^

Collectively, our results highlight that ETI treatment promoted phenotypic features of a metabolically switched Cluster-2 with enhanced expression of PD-L1, potentially reflecting profound modifications in neutrophil plasticity and presumably potentiating immunosuppressive functions in CF neutrophils.

### FPR1^high^/CXCR2^high^ and PD-L1^high^/CD114^high^/GLUT-1^high^ neutrophil subsets enrichment differentially correlate with clinical parameters in pwCF

**Figure 6:**
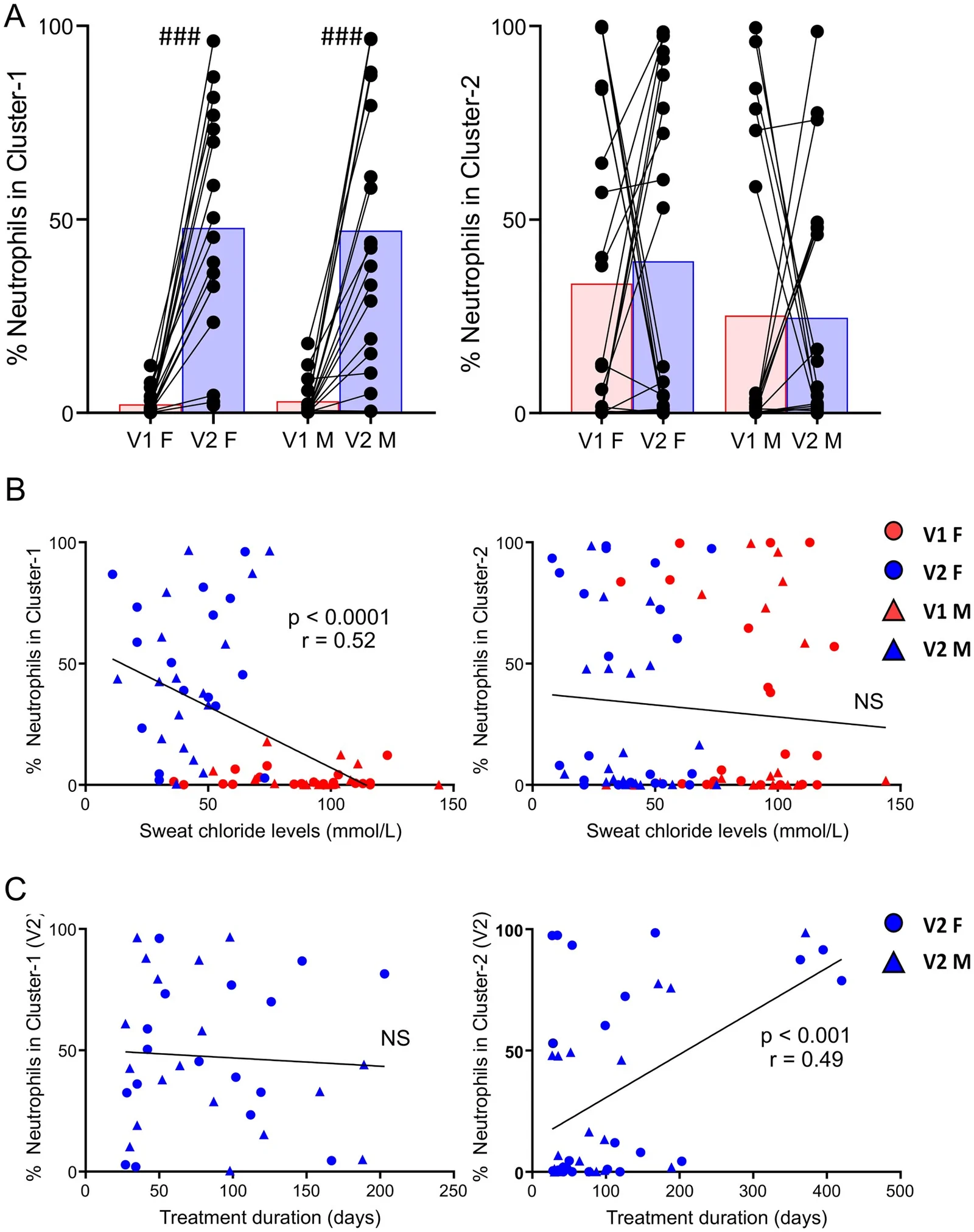
Analysis of the impact of sex, sweat chloride levels and treatment duration on FPR1^high^/CXCR2^high^ and PD-L1^high^/CD114^high^/GLUT-1^high^ neutrophil subsets enrichment. (A) Analysis of FPR1^high^/CXCR2^high^ (Cluster-1) and PD-L1^high^/CD114^high^/GLUT-1^high^ (Cluster-2) neutrophil subsets enrichment according to the biological sex (female, F, or male, M) of pwCF before (V1) and after (V2) ETI treatment. Statistical differences between groups were calculated using either unpaired Mann–Whitney test to compare male and female, or paired Wilcoxon test to compare V1 and V2 (### *P* < 0.001). (B) Analysis of the correlation between Cluster-1 or Cluster-2 neutrophils enrichments and sweat chloride levels in V1 male pwCF (red triangles), V2 male pwCF (blue triangles), V1 female pwCF (red circles) and V2 female pwCF (blue circles). Correlation coefficients calculated using linear regression and *P* values using the Student *t* test are displayed. (C) Analysis of the correlation between Cluster-1 or Cluster-2 neutrophils enrichments and treatment duration for V2 male pwCF (blue triangles) and V2 female pwCF (blue circles). Correlation coefficients calculated using linear regression and *P* values using the Student *t* test are displayed.

Given that sex differences in neutrophil biology have previously been described in the literature,^42^ we assessed whether the enrichment of FPR1^high^/CXCR2^high^ and PD-L1^high^/CD114^high^/GLUT-1^high^ neutrophils in pwCF could be influenced by biological sex. Here, we did not detect any sex-driven differences in neutrophil subset enrichment (Fig. 6A).

Instead, we identified sex-dependent changes in marker expression levels in untreated and treated pwCF (Fig. S6). PD-L1 and CD64 expression levels at V1 were significantly higher in males than in females. CD63 and CD16 expression significantly increased at V2 in female pwCF but not in males, whereas CD11c expression decreased at V2 in males and remained unchanged in females.

The clinical benefits of ETI treatment and improved CFTR functionality have been associated with decreased chloride levels in the sweat of pwCF, commonly referred to as the sweat chloride test.^27^ Accordingly, the enrichment of FPR1^high^/CXCR2^high^ neutrophils in V1 and V2 negatively correlated with sweat chloride levels, with no difference between males and females (Fig. 6B), indicating that restoration of this subset after treatment may be associated with CFTR correction. However, the enrichment of FPR1^high^/CXCR2^high^ neutrophils did not correlate with treatment duration (Fig. 6C), another important clinical variable in the present study.

In contrast, the enrichment of PD-L1^high^/CD114^high^/GLUT-1^high^ neutrophils did not correlate with sweat chloride levels but correlated positively with treatment duration, again with no difference between males and females.

Of note, there was no correlation between treatment duration and activation marker levels, except for CD63, whose expression was positively correlated with treatment duration (Fig. S7).

The enrichment of these neutrophil subsets did not correlate with age (data not shown) or with the percent predicted forced expiratory volume in one second (ppFEV_1_), an indicator of respiratory function (Fig. S8). Collectively, these results highlight the importance of considering the impact of these clinical variables on neutrophil phenotype and subset composition.

## Discussion

Neutrophils drive the bulk of inflammation-mediated pulmonary disease in CF and thus are logical targets for therapeutic intervention. By leveraging state-of-the-art spectral cytometry, we discovered that circulating neutrophils in pwCF before ETI treatment are heterogenous, which presumably reflects differential rates of mobilization from bone marrow, differentiation, and activation in response to cytokines and inflammatory mediators.^11^ Furthermore, unsupervised Vi-tSNE analysis identified 2 distinct neutrophil subsets that may differ functionally, namely the pathogen sensing related Cluster-1, characterized by FPR1^high^/CXCR2^high^ neutrophils and the metabolism/immunomodulation-related Cluster-2, characterized by PD-L1^high^/CD114^high^/GLUT-1^high^ neutrophils.^28^^,^^29^ We found that rather than producing uniform changes across the entire neutrophil population, ETI induced alterations in expression of phenotypic markers of maturation, pathogen sensing, and immunomodulation that are specific to particular neutrophil subsets. The identification of distinct subpopulations of neutrophils in untreated pwCF and the notation of phenotypic changes temporally associated with ETI treatment underscore the clinical potential of our findings.

Regarding neutrophil activation state, our data confirm a decrease in degranulation markers (CD63 and CD66b) in neutrophils from pwCF that ETI treatment normalized. Indeed, previous studies described a dysregulation in granule trafficking and in primary and secondary degranulation, including CD66b expression, in neutrophils from pwCF directly or indirectly linked to CFTR dysfunction.^30^ Moreover, the lack of significant modulation of the expression of the phagocytic receptor CD64 after ETI treatment could be related to the persistence of infection, as previously reported in sepsis.^31^

Regarding neutrophil maturity, circulating CF neutrophils obtained prior to ETI treatment exhibited reduced expression of differentiation markers compared with HD neutrophils, consistent with previous studies.^32^^,^^33^ However, ETI treatment did not fully restore a normal maturity-associated phenotypic profile similar to that of HD, as CD10 expression remained lower in the pathogen-sensing Cluster-1, highlighting a subset-specific effect. Whether ETI directly influences neutrophil differentiation remains an open question. Notably, RNA interference against CFTR^34^^,^^35^ or expression of the deltaF508mutant in the promyelocytic HL-60 cell line^18^ shows no effect on granulocytic differentiation, leaving the precise mechanism unresolved.

Nonetheless, we observed a full restoration of the FPR1^high^/CXCR2^high^ neutrophil subset associated with Cluster-1, which was almost totally absent in CF neutrophils before treatment. Upregulation of FPR1 and CXCR2, which is required for the response to IL-8, delineated a neutrophil subset that might be involved in attributes beneficial to the host, such as pathogen sensing and chemotaxis. Restoration of FPR1 surface expression may suggest an increased expression of CFTR after ETI, since CFTR redistribution to cell surface correlates with the recruitment of FPR1 during granule exocytosis.^35^ In line with this, we have observed a negative correlation of the FPR1^high^/CXCR2 ^high^ subset enrichment with the sweat chloride levels of pwCF, indicating that the restoration of the subset after treatment was associated with the correction of CFTR functionality. Evidence for improved pathogen sensing and antimicrobial phenotype positions the FPR1^high^/CXCR2^high^ subset as a potential biomarker for ETI-driven immune reprogramming.

Cluster-2 characterized a subset with remodeled metabolic and immunosuppressive phenotype in CF neutrophils, which is associated with increased expression of the immune checkpoint PD-L1, the glucose transporter GLUT-1, and the G-CSF receptor CD114, all of which were increased after initiation of ETI therapy. Expression of CD114 within Cluster-2 suggests that neutrophils might be responsive to G-CSF, which could drive a metabolic rewiring with potentiation of the glycolytic pathway, as previously demonstrated.^36^ This relationship is corroborated by previous studies showing increased G-CSF level in plasma from pwCF.^37^ Neutrophils within this subset upregulated expression of CD10 and CD45 after ETI treatment, demonstrating that they were fully mature.^12^^,^^32^ Moreover, the strongly increased expression of integrins such as CD11b and CD11c suggests an ability of neutrophils in this cluster to perform integrin-dependent cross-talk with other immune cells, an activity that might contribute to T cell mediated immunosuppressive activities, as previously described.^38^

The Cluster-2 present in HD was expanded in CF and persisted at similar levels after ETI treatment, as opposed to the FPR1^high^/CXCR2^high^ subset that was disrupted in CF and restored after ETI treatment. We identified a dual pattern of Cluster-2 enrichment in CF and after ETI treatment, with some pwCF exhibiting increased whereas others showing decreased Cluster-2 enrichment following ETI initiation. This effect was not associated with age, sex, treatment duration, sweat chloride levels or ppFEV_1_ (data not shown).

By using a 13-marker spectral flow cytometry panel on another cohort of untreated pwCF with a stable clinical status or presenting pulmonary exacerbations, we have previously identified by t-SNE analysis the expansion of a similar (possibly the same as here) CD114^high^/PD-L1^high^ subset that was expanded in CF.^13^ This subset expansion was not modulated by clinical exacerbations.

Collectively, our analysis of the different neutrophil subsets in the blood stream compartment raises critical questions regarding the selective recruitment of specific neutrophil subsets to the lung and the extent to which ETI might modulate neutrophil dynamics. Lung neutrophils from CF undergo metabolic reprogramming and show a significant increase in GLUT-1 expression compared to controls,^39^^,^^40^ degranulation, immunosuppressive abilities and arginase expression leading to resistance to apoptosis.^41^

The inclusion prospectively of a relatively large number of pwCF at the critical time of ETI initiation represents a main strength of this study and provides a unique opportunity to examine the effectiveness of CFTR modulator combinations on neutrophil functional immunophenotype. Nonetheless, our study has some limitations: we did not perform any functional assessment on the specific subsets that we have identified. For example, we did not assess the bactericidal activity and immunosuppressive capacities of the FPR1^high^/CXCR2 ^high^ and PD-L1^high^/CD114^high^/GLUT-1^high^ neutrophil subsets, respectively. We also recognize that our observations of the heterogeneity of circulating neutrophils focused on a limited time interval (1 to 12 mo) of exposure to ETI, and it remains unknown whether longer exposure to ETI would result in additional changes in neutrophil plasticity or a return to a normal pattern. However, we observed that the difference in CD63 expression between untreated and treated pwCF positively correlated with treatment duration (Fig. S7). The enrichment of PD-L1^high^/CD114^high^/GLUT-1^high^ neutrophils in treated versus untreated pwCF also correlates with treatment duration (as opposed to the FPR1^high^/CXCR2^high^ subset), suggesting that the effects of ETI therapy on neutrophils may be dynamic over time (Fig.6C). Nevertheless, there remains no evidence that the changes in surface marker expressions that we observed are durable.

The limited information on clinical characteristics of HD (age and gender) due to the anonymous nature of blood donation in France is an unavoidable limitation of clinical studies such as ours. Nonetheless, given that sex differences in neutrophil biology have already been described in the literature,^42^ we identified sex-driven changes in marker expression levels (but not in subset enrichment) in untreated and treated pwCF (Fig. S6). These findings highlight the importance of considering sex as a key biological variable when investigating neutrophil dysfunction in disease and in the development of adjunctive therapies.

Finally, our findings leave unanswered whether ETI exerted its effects on neutrophils through direct modulation of CFTR or via CFTR-independent pathways, a distinction with significant therapeutic implications. Addressing this question requires experimental models, such as F508del-CF HL-60 cells, that can differentiate into mature CF neutrophils.^18^ Such model cells exhibit a pro-inflammatory basal state and an aberrant response to LPS, strongly suggesting that CFTR dysfunction is causally responsible for the functional abnormalities observed in CF. Whether ETI can directly target CFTR in neutrophils currently remains unknown.

In conclusion, elucidating the evolution of circulating neutrophil plasticity will be crucial to understand the long-term effects of ETI, to evaluate their impact on the neutrophil maturation and functions and more globally on the immune response to refine anti-inflammatory strategies in CF. Adjunctive therapies that target neutrophil metabolic and functional plasticity could enhance beneficial antimicrobial subsets while simultaneously mitigating immunosuppressive phenotypes, thereby opening new perspectives for immune modulation in CF treatment.

## Supplementary Material

vlag030_Supplementary_Data

## Data Availability

All raw flow cytometry data have been deposited in the Institut Cochin data repository (CID Cochin Database, HAL ID: hal-04834159v2) and are publicly available via DOI: https://doi.org/10.57889/kj9h-2m84. The dataset includes raw spectral (non-unmixed) FCS files for all individuals in HD, V1, and V2 groups, as well as corresponding unstained and single-stained controls used for spectral unmixing. These files enable independent reconstruction of the unmixing matrix and full reanalysis of the data.
