# Supplementary figures and images for: CFTR modulators exert subset-specific phenotype remodeling on circulating neutrophils in cystic fibrosis
Source: Immunohorizons. 2026 Jul 31;10(7):vlag030. doi: 10.1093/immhor/vlag030 (PMC13426474; doi:10.1093/immhor/vlag030)

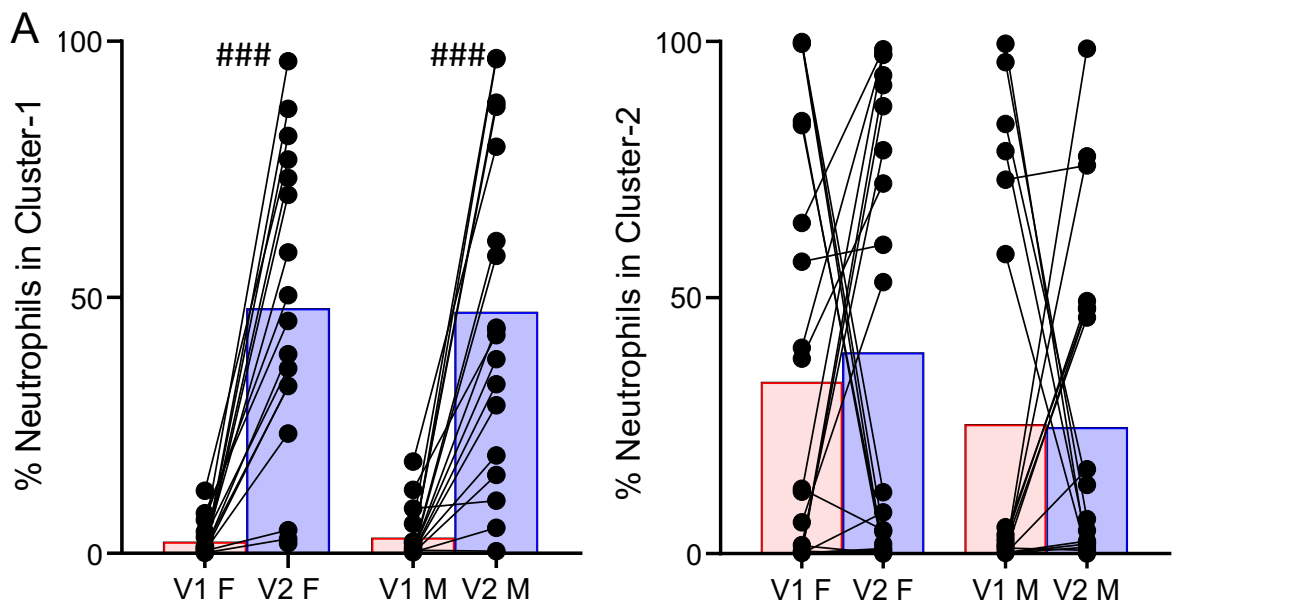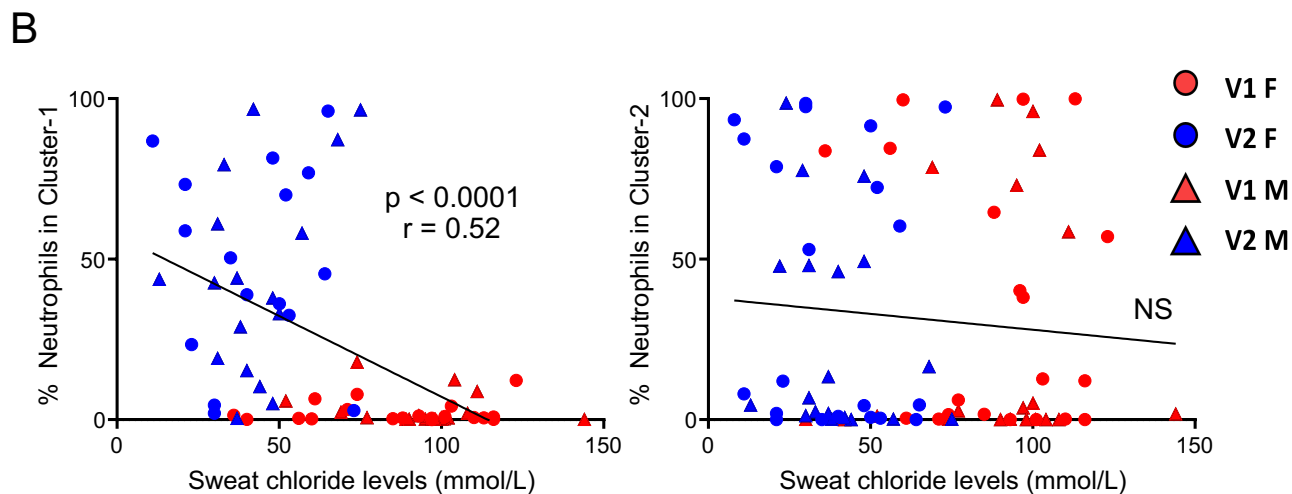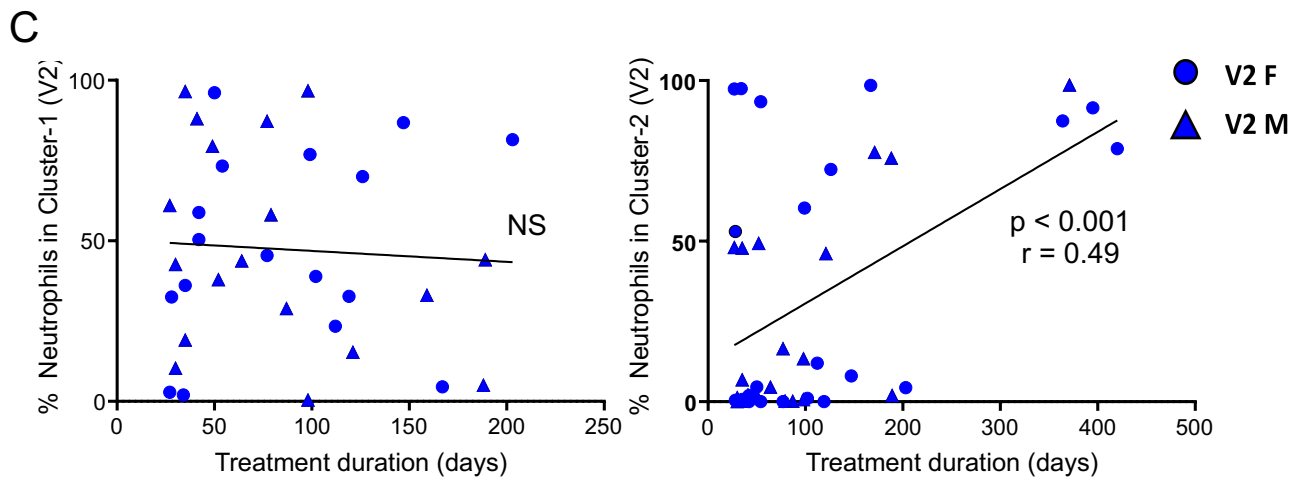

Supplement: vlag030_Supplementary_Data [file vlag030_supplementary_data.zip › Corrected-Figures_6_with_#.pdf]
