# Supplementary material for: CFTR modulators exert subset-specific phenotype remodeling on circulating neutrophils in cystic fibrosis
Source: Immunohorizons. 2026 Jul 31;10(7):vlag030. doi: 10.1093/immhor/vlag030 (PMC13426474; doi:10.1093/immhor/vlag030)
Supplement: vlag030_Supplementary_Data [file vlag030_supplementary_data.zip › Supplementary_Data.zip!/Chable_de_la_Heronniere_et_al-supplementary material-ImmunoHorizons.pdf]

ONLINE Supplementary material

CFTR modulators exert subset-specific phenotype remodeling on circulating neutrophils in cystic fibrosis

Short title: Neutrophil heterogeneity in cystic fibrosis

François Chable de la Héronnière[*,†], Théo Dhôte[*,†,‡,§], Rodrigo de Oliveira Formiga[*,†], Lucile Regard[*,†,‡,§], Giovanni Saraceni Tasso[*,†], Muriel Andrieu[*,†], Souganya Many[*,†], Jennifer Da Silva[*,†,‡,§], Frédéric Pène[*,†,¶], Clémence Martin[*,†,‡,§], Maha Zohra Ladjemi[*,†], Pierre-Régis Burgel[*,†,‡,§], Véronique Witko-Sarsat[*,†]

Affiliations:

* Inserm U1016, Institut Cochin, CNRS UMR8104, Paris, France

† Université Paris-Cité, Paris, France

‡ Service de Pneumologie & Centre de Référence Maladies Rares Mucoviscidose, site coordonnateur, Hôpital Cochin, Assistance Publique-Hôpitaux de Paris-Centre & Université Paris-Cité, Paris, France

§ERN-Lung, cystic fibrosis core network, Frankfurt, Germany

¶ Service de Médecine intensive & Réanimation, Hôpital Cochin, Assistance Publique-Hôpitaux de Paris-Centre & Université Paris-Cité, Paris, France

Corresponding author:

Véronique Witko-Sarsat, Inserm U1016, Institut Cochin, 27 rue du Faubourg Saint Jacques, F75014 Paris, France; E-mail: veronique.witko@inserm.fr; Phone: +33140516656

This PDF file contains:

- **Supplementary table S1**
- **Supplementary figures S1 to S8**

I/ Supplementary table

	Antigen	Full names	Fluorochrome	Clone	Supplier
Maturity	CD45	Protein tyrosine phosphatase receptor type C	PerCP	2D1	BioLegend
	CD33	Sialic acid binding Ig-like (lectin 3, Siglec-3, gp67, p67)	BUV-615	P67.6	BD Biosciences
	CD13	aminopeptidase N, membrane alanyl aminopeptidase	PerCP-Cy5.5	WM15	BioLegend
	CD16	Fc gamma RIII	AF-700	3G8	BioLegend
	CD10	Neprilysin, common acute lymphoblastic leukemia antigen, neutral endopeptidase	BUV-737	HI10α	BD Biosciences
	CD15	Lewis x trisaccharide	BUV-805	W6D3	BD Biosciences
Activation	CD64	Fc gamma RI	BV-480	10.1	BD Biosciences
	CD14	Monocyte differentiation antigen CD14, Lipopolysaccharide co-receptor CD14	APC-H7	MφP9	BD Biosciences
	FPR1	Formyl-peptide receptor 1	APC	W15086B	BioLegend
	CXCR2	C-X-C Motif Chemokine Receptor 2, Interleukin-8 receptor beta (IL-8Rβ), CD182	BUV-496	6C6	BD Biosciences
Bactericidy	CD62L	L-selectin, Leukocyte adhesion molecule 1 (LAM-1),	BV-785	DREG-56	BioLegend
	CD11b	Integrin alpha M, Complement receptor 3 alpha subunit (CR3α)	BUV-395	ICRF44	BD Biosciences
	CD11c	Integrin alpha X, p150 alpha chain, Complement receptor 4 alpha subunit (CR4α)	AF-647	3.9	BioLegend
	CD63	Tetraspanin 30, LAMP-3–associated protein, Granulophysin	PE-Cy7	H5C6	BioLegend
	CD66b	Carcinoembryonic antigen-related cell adhesion molecule 8 (CEACAM8), CGM6	PE-Dazzle-594	G10F5	BioLegend
	CD177	NB1 glycoprotein, Human Neutrophil Antigen-2a (HNA-2a)	BUV-563	MEM-166	BD Biosciences
Immunomodulation and metabolism	LOX-1	Lectin-like oxidized low-density lipoprotein receptor 1 (OLR1)	PE	15C4	BioLegend
	GLUT-1	Glucose transporter type 1	AF-488	202915	RD Systems
	CD114	Granulocyte Colony-Stimulating Factor Receptor, Colony Stimulating Factor 3 Receptor (CSF3R)	BV-510	LMM741	BD Biosciences
	PD-1	Programmed Cell Death Protein 1	BUV-661	EH12.1	BD Biosciences
	PD-L1	Programmed Death-Ligand 1, CD274, B7-H1	BV-421	MIH3	BioLegend
Inhibitory receptors	Siglec-5	Sialic acid-binding immunoglobulin-like lectin 5	BV-711	1A5	BD Biosciences
	Siglec-8	Sialic acid-binding immunoglobulin-like lectin 8	PE-Cy5	7C9	BioLegend
	Siglec-9	Sialic acid-binding immunoglobulin-like lectin 9	BV-605	E10-286	BD Biosciences

Supplementary table S1: List of antibodies used for neutrophil immunophenotyping. The antibodies used for spectral flow-cytometry staining are indicated, including their specificity, associated fluorochrome, clone and supplier. Markers are classified according to neutrophils biological functions. CD45 is a leukocyte lineage marker; CD15, CD16 (FcγRIII) and CD10 are granulocytic maturity markers; CD13 is an aminopeptidase indicative of neutrophil differentiation; CD33 (Siglec 3) is an inhibitory receptor; CD14 and FPR1 are involved in bacterial recognition; CXCR2 mediates IL-8-driven neutrophil migration; CD64 (FcγRI) is involved in phagocytosis; CD11b, CD11c are adhesion molecules; CD62L, CD63 and CD66b are degranulation markers; CD177 is an activation marker; PD-1 (programmed death-1) and PD-L1 (programmed death-ligand 1) are immunosuppressive checkpoint molecules; LOX1 (lectin-like oxidized low-density lipoprotein receptor-1) and GLUT-1 (glucose transporter-1) are the metabolic receptors; CD114 is the G-CSF receptor; Siglec-5, Siglec-8, and Siglec-9 are inhibitory sialic acid receptors.

II/ Supplementary figures

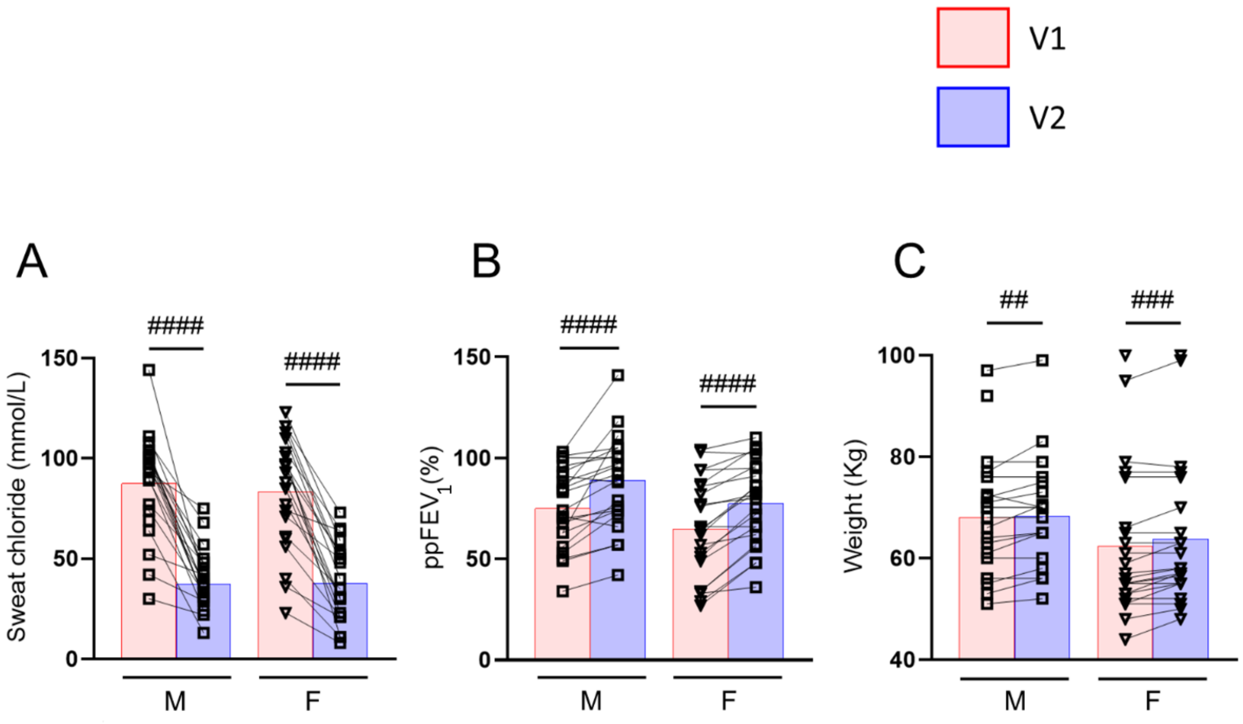

Supplementary figure S1: Effect of ETI on clinical parameters according to the sex at birth of pwCF. (A) Measurement of chloride concentration in sweat from pwCF. (B) Measurement of percent predicted forced expiratory volume in one second ($ppFEV_1$). (C) Evaluation of weight. Individual data is shown for each subject and group means are represented with bars for untreated male and female pwCF (V1 M and V1 F) and under ETI treatment (V2 M and V2 F). Statistical analysis has been performed either using unpaired Mann-Whitney test when comparing V1 M vs V1 F and V2 M vs V2 F or using paired Wilcoxon test when comparing V1 M vs V2 M and V1 F vs V2 F (##$p < 0.01$, ###$p < 0.001$, ####$p < 0.0001$).

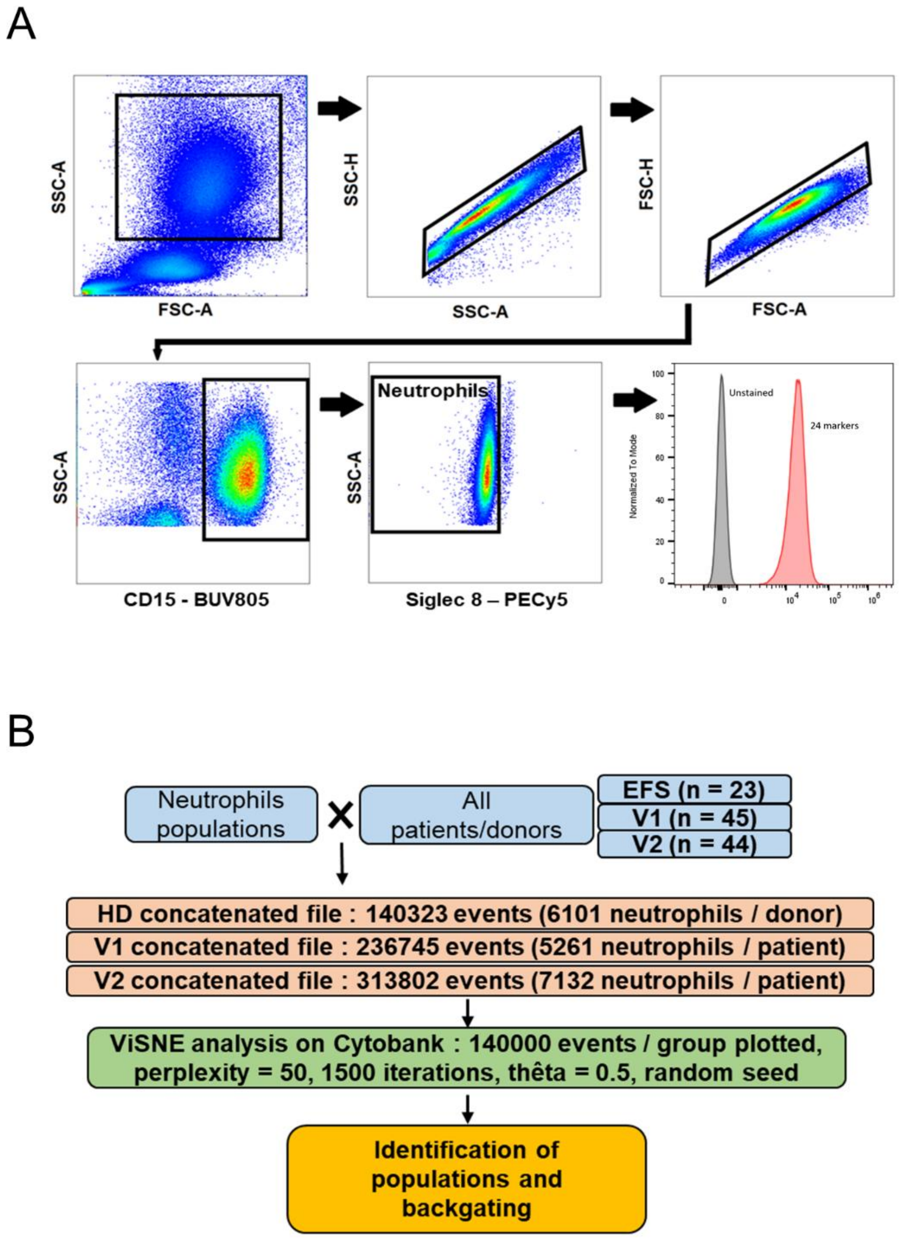

Supplementary figure S2: Gating strategies and methods for supervised and unsupervised flow cytometry analysis. (A) Gating strategy for neutrophil identification and immunophenotyping within whole blood. Granulocytes were first selected by size (FSC) and granularity (SSC). After exclusion of doublets (SSC-H/SSC-A and FSC-H/FSC-A), CD15 positive cells resulted in the neutrophil population. Finally, residual eosinophils were excluded using their higher Siglec 8 expression. The panel of 24 markers is detailed in Supplementary Table 1. Expression of each individual surface marker was expressed by the mean fluorescence intensity (MFI). (B) Strategy and parameters used to represent flow cytometry data with Visual interactive t-distributed Stochastic Neighbor Embedding (Vi-tSNE) dimensional reduction analysis. A sample of events was randomly taken from each patient (HD: 6101/donor, V1: 5262/patient, V2: 7132/patient). All events were then grouped into concatenated files (HD: 140323 events, V1: 236745 events, V2: 313802 events). Using Cytobank online platform (Beckman Coulter Life Sciences, Brea, US), a total of 140000 random events for each group were then subjected to the algorithm to form clusters according to the expression levels of the surface phenotyping markers, using a rainbow heat scale to represent expression intensity, from red indicating high expression levels to blue indicating low expression levels. For every individual (either HD or pwCF), cluster identity was systematically verified by backgating and the individual enrichment of clusters among total neutrophils was reported.

A

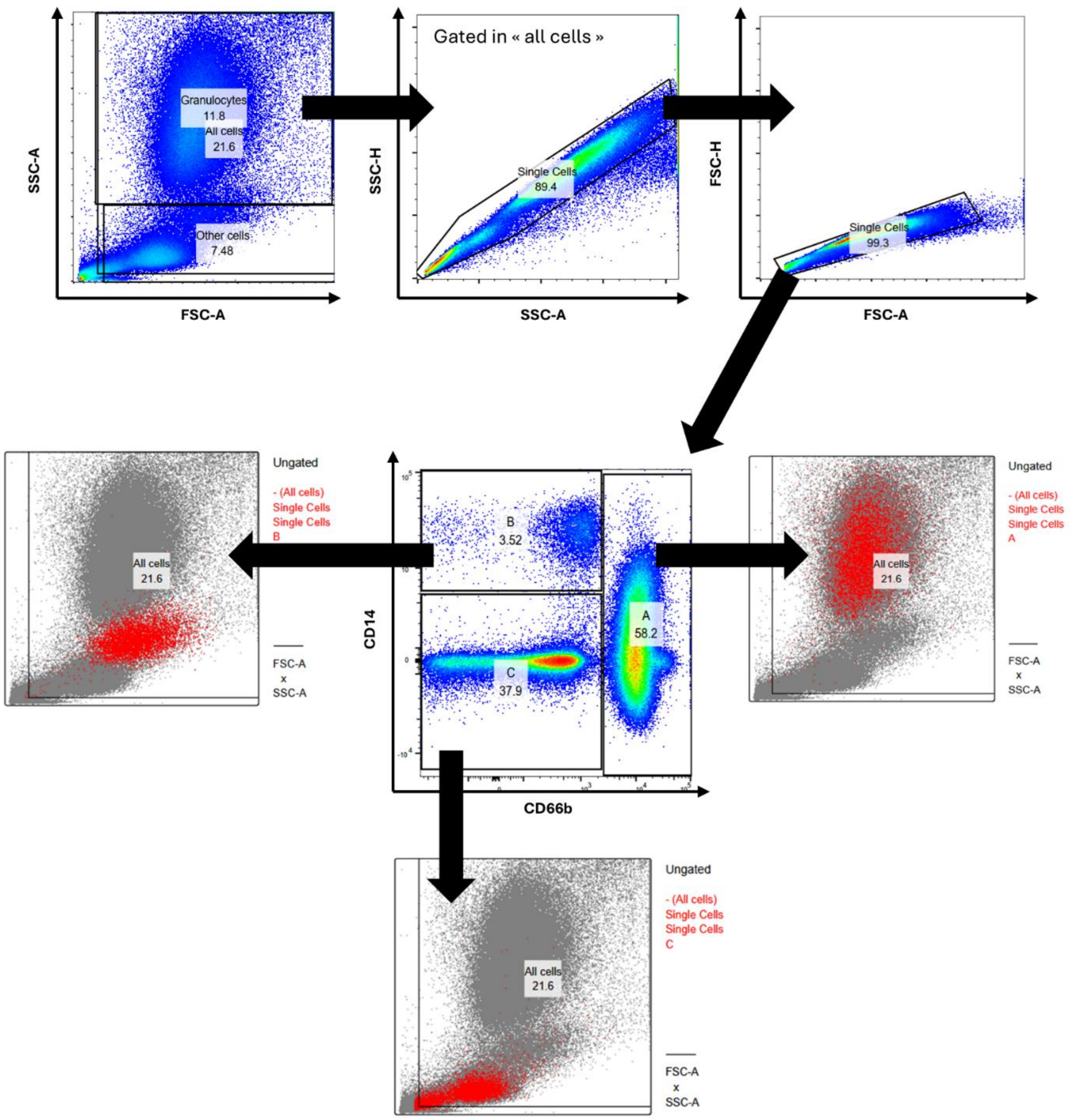
SSC-A
FSC-A
Granulocytes
11.8
All cells
21.6
Other cells
7.48
Gated in « all cells »
SSC-H
SSC-A
Single Cells
89.4
FSC-H
FSC-A
Single Cells
99.3
CD14
CD66b
B
3.52
A
58.2
C
37.9
Ungated
- (All cells)
Single Cells
Single Cells
B
A
C
FSC-A
x
SSC-A

B

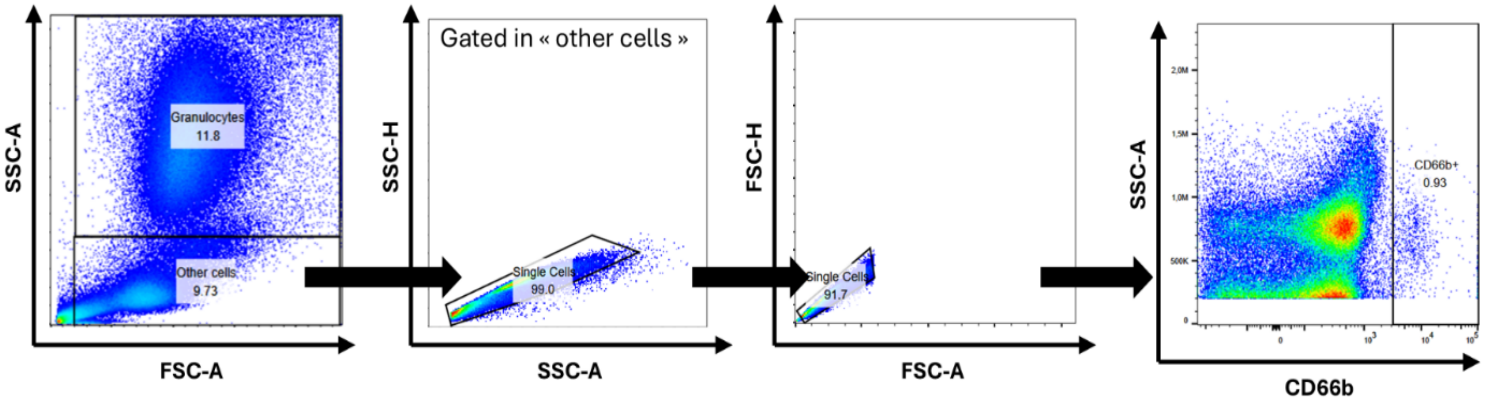
SSC-A
FSC-A
Granulocytes
11.8
Other cells
9.73
Gated in « other cells »
SSC-H
SSC-A
Single Cells
99.0
FSC-H
FSC-A
Single Cells
91.7
SSC-A
CD66b
CD66b+
0.93

C

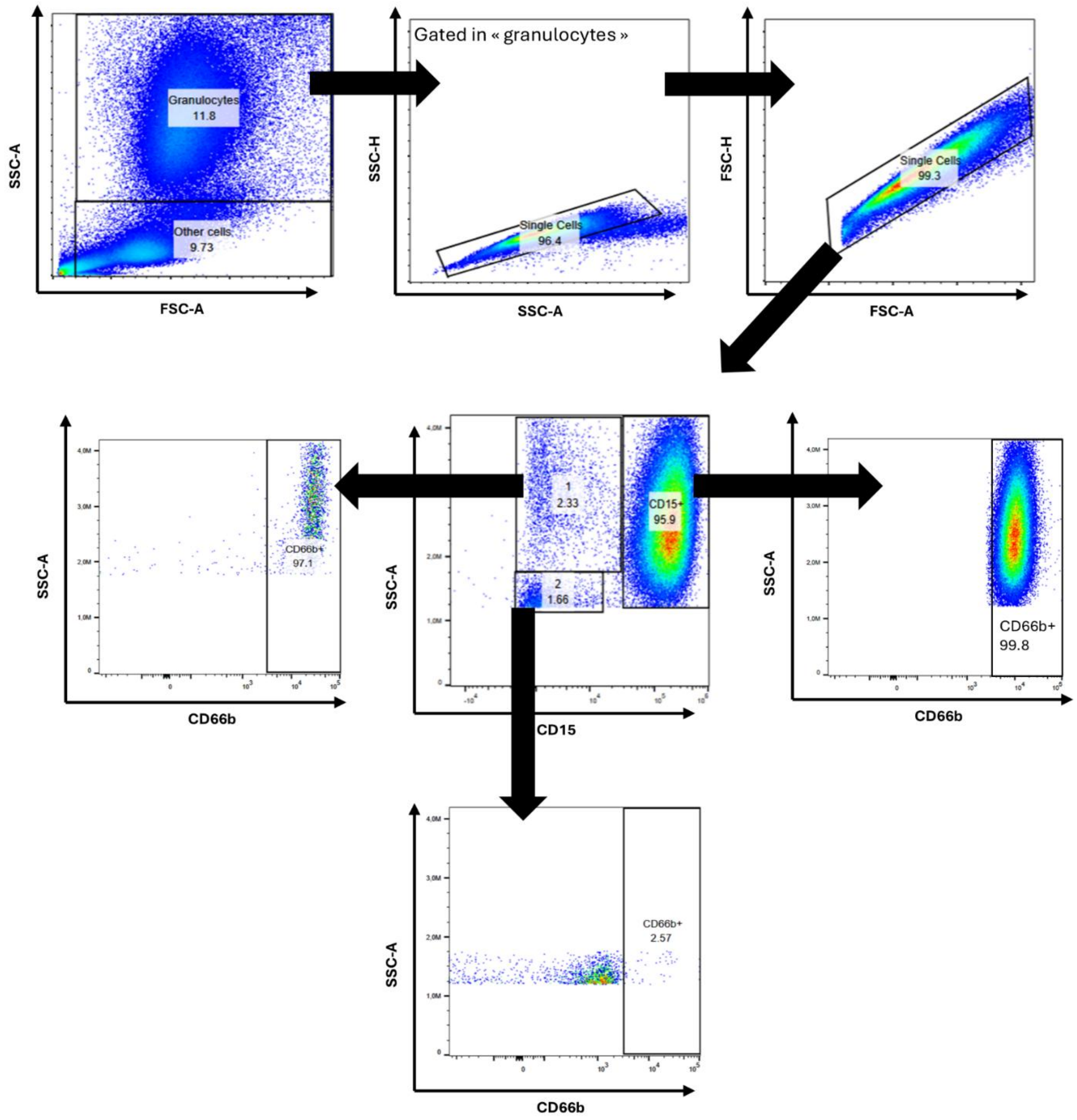

Supplementary figure S3: Analysis of neutrophil specificity of the whole-blood flow cytometry gating strategy. (A) Backgating analysis was performed to verify the neutrophil specificity of our gating strategy in whole blood. To this end, two gates were initially defined in the SSC/FSC plot: a classical "granulocyte" gate used in our analysis and a second gate containing the remaining cells ("other cells"). An additional "all cells" gate was created, while debris were systematically excluded. After doublet exclusion, events from the "all cells" gate were projected according to CD66b and CD14 expression. CD66b was used as a specific marker for neutrophils, whereas CD14 enabled discrimination between granulocytes (moderate expression), monocytes (higher expression), and lymphocytes or NK cells (CD14 negative). This analysis identified three populations: a CD66b^{+} population ("A") representing approximately 60% of all gated events, consistent with the expected proportion of granulocytes among leukocytes, and two CD66b^{-} populations ("B" and "C"). Population "B" displayed high CD14 expression and represented about 3.5% of leukocytes, consistent with monocytes, whereas population "C" was CD14^{-}. Backgating of these populations in the SSC/FSC plot revealed distinct distributions: events in population "A" corresponded to CD66b^{+} granulocytes, those in "B" to CD66b^{-}/CD14^{+} monocytes, and those in "C" to CD66b^{-}/CD14^{-} cells, corresponding to lymphocytes and NK cells. Notably, population "A" precisely overlapped with the granulocyte gate used for neutrophil identification. These results demonstrate that our size-

based gating strategy selectively captures CD66b$^+$ granulocytes while excluding CD66b$^-$ monocytes without detectable loss of neutrophils. The same analysis was repeated using CD33 instead of CD14 and yielded identical results. (B) Within the "other cells" SSC/FSC gate, less than 1% of events were CD66b$^+$, confirming the high specificity of the granulocyte gate for neutrophils. (C) Additional analysis was performed on the CD15$^-$ fraction remaining after selection of CD15$^+$ cells within the granulocyte SSC/FSC gate. As expected, the CD15$^+$ gate was CD66b$^+$. A small population of CD15$^-$ cells with high SSC (gate "1") was also CD66b$^+$ and represented approximately 2% of granulocytes. These cells were excluded and may correspond to immature neutrophils. A second population ("2") corresponded to a small fraction of the previously identified CD14$^+$/CD66b$^-$ gate and likely represents residual monocytes. These complementary analyses were performed on a representative healthy donor and showed similar results in untreated and ETI-treated pwCF.

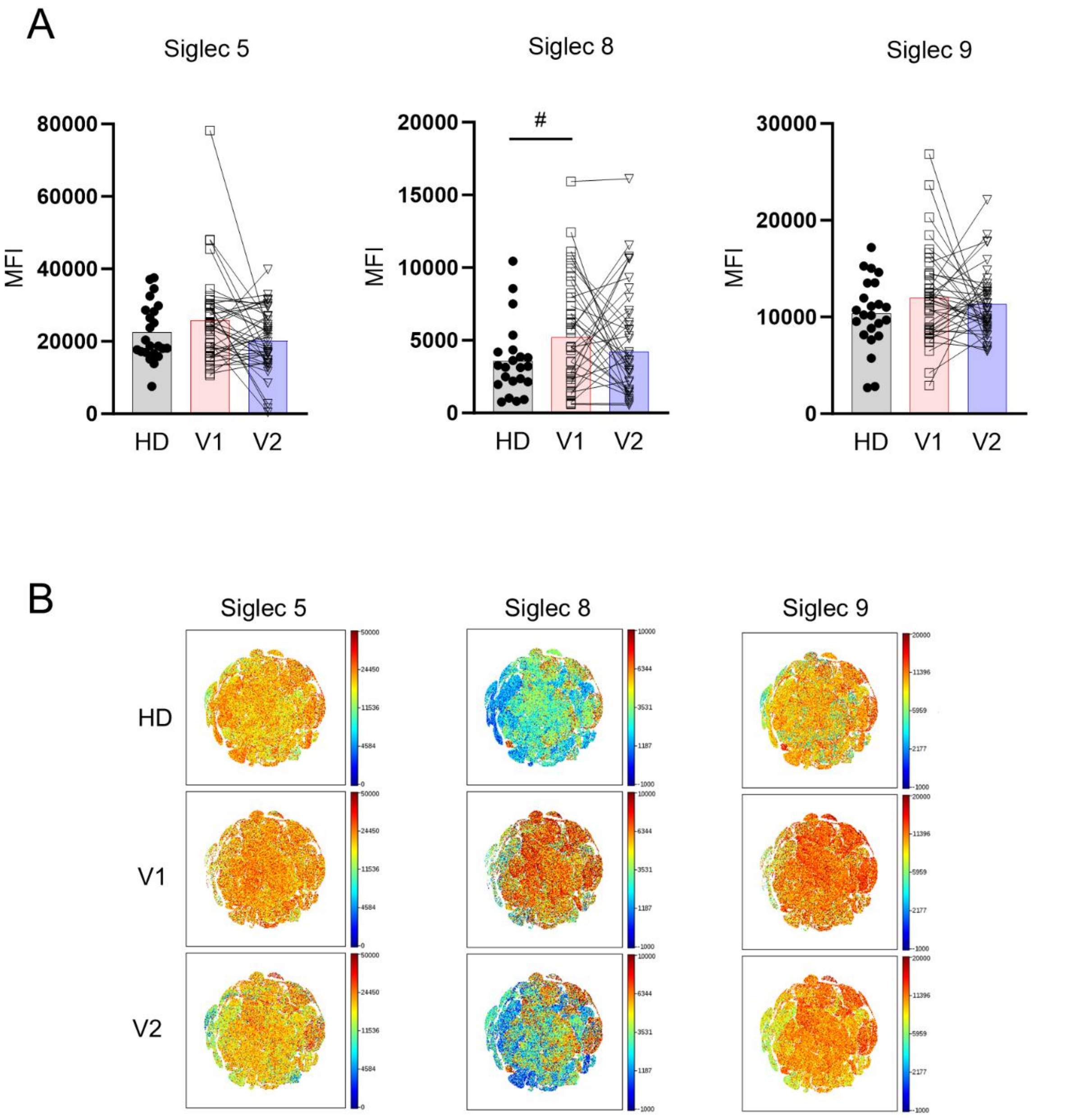

Supplementary figure S4: Analysis of ETI treatment effect on the expression of Siglec 5, Siglec 8 and Siglec 9. (A) Mean Fluorescence Intensities (MFI) are represented for Siglec 5, Siglec 8 and Siglec 9 surface markers of circulating neutrophils from healthy donors (HD), untreated patients (V1) and patients under ETI treatment (V2). Individual data is shown for each subject and group medians are represented with bars. Statistical analysis has been performed either using unpaired Mann-Whitney test when comparing HD vs V1 and HD vs V2 or using paired Wilcoxon test when comparing V1 vs V2 ($p < 0.05$). The p-values were corrected for multiple comparison using the false discovery rate (FDR) method. (B) Visualization of expression clusters for Siglec 5, Siglec 8 and Siglec 9 on neutrophils from HD, V1 and V2, using Visual interactive t-Distributed Stochastic Neighbor Embedding (Vi-tSNE). Plots are represented following a rainbow heat scale for median fluorescence intensity, from red indicating high expression levels to blue indicating low expression levels.

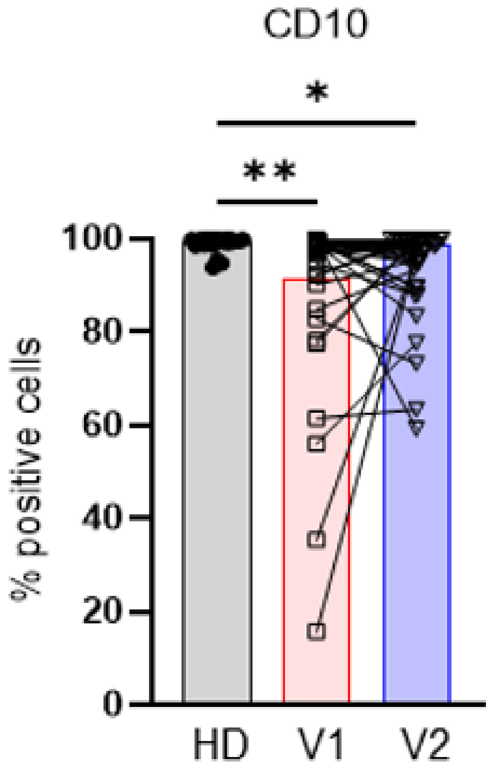

Supplementary figure S5: Analysis of the percentages of CD10^{+} neutrophils. Analysis of the percentage of CD10+ neutrophils in healthy donors (HD), untreated (V1) and ETI-treated pwCF (V2). Individual data is shown for each subject and group means are represented with bars. Statistical analysis has been performed either using unpaired Mann-Whitney test when comparing HD vs V1 and HD vs V2 (*$p < 0.05$; **$p < 0.01$) or using paired Wilcoxon test when comparing V1 vs V2.

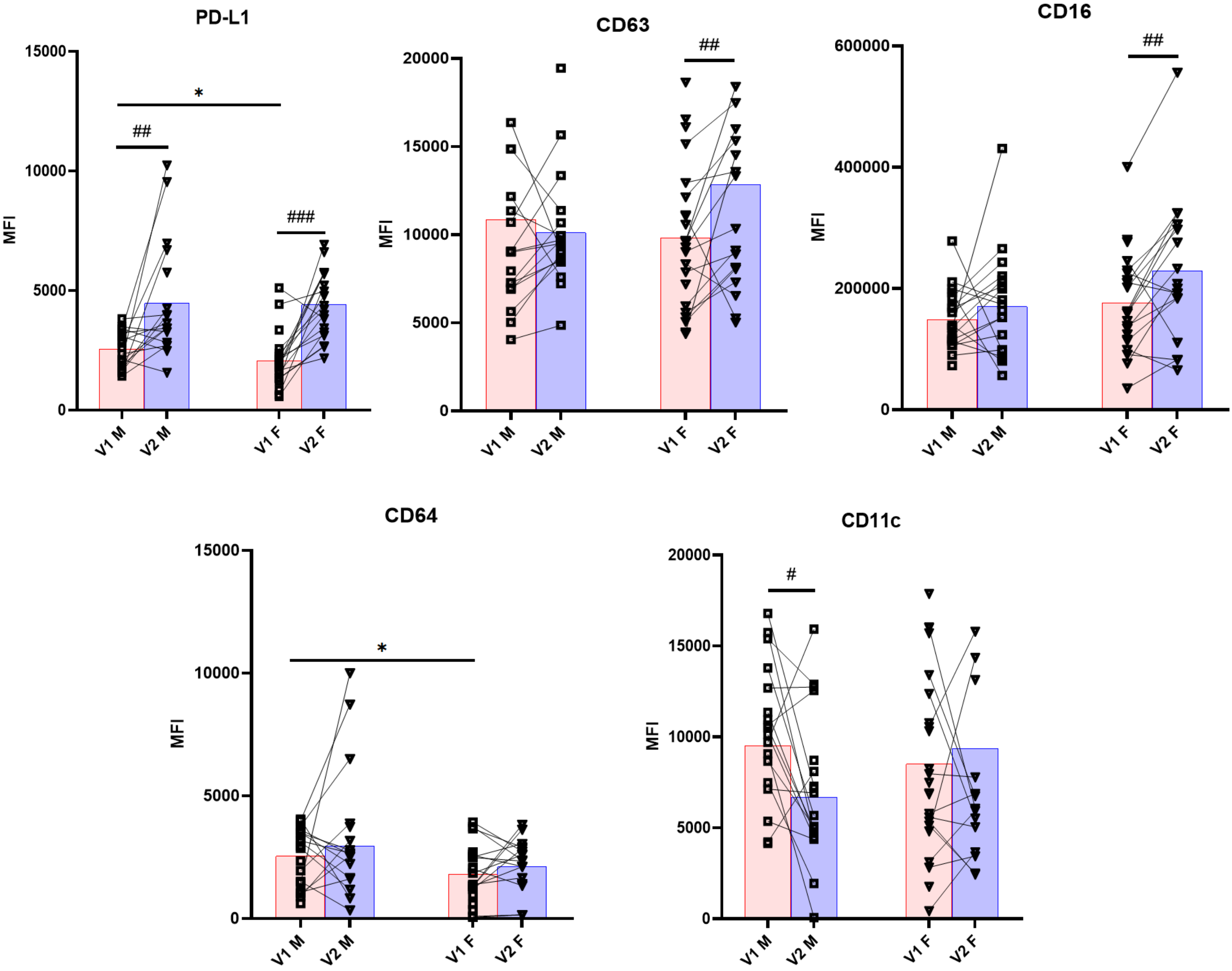

Supplementary figure S6: Analysis of sex-driven differences in marker expression in neutrophils from pwCF. Analysis of individual surface marker expressions of PD-L1, CD63, CD16, CD64 and CD11c in whole blood circulating neutrophils from healthy donors (HD), untreated (V1) and ETI-treated (V2) pwCF by separating male and female individuals. Data are Mean Fluorescence Intensities (MFI) measured for each subject and group medians are represented with bars. Statistical analysis has been performed either using unpaired Mann-Whitney test when comparing HD vs V1, HD vs V2 and male vs female (*p < 0.05) or using paired Wilcoxon test when comparing V1 vs V2 (#p < 0.05; ##p < 0.01; ###p < 0.001). The *p*-values were corrected for multiple comparison using the false discovery rate (FDR) method.

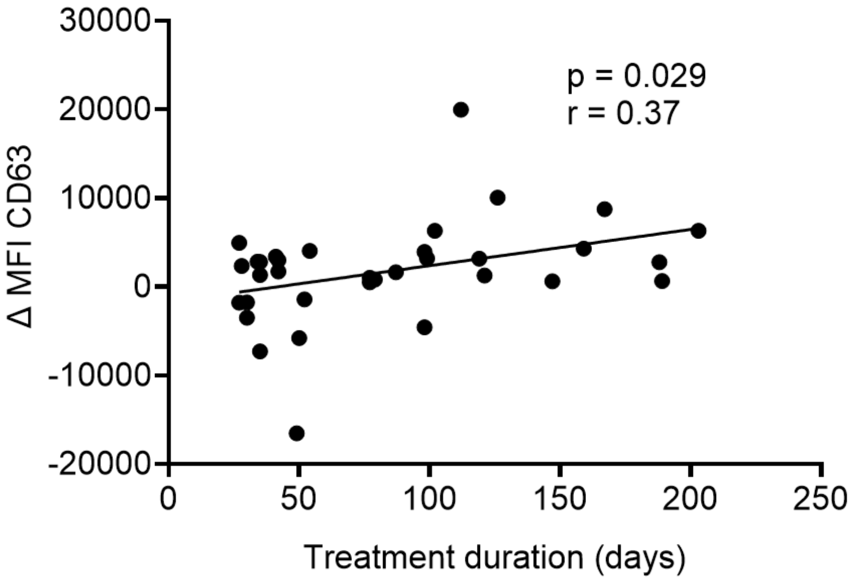

Supplementary figure S7: Effect of treatment duration on CD63 expression levels in neutrophils from pwCF. Correlation between the treatment duration and the difference in CD63 expression levels in neutrophils from pwCF (V2-V1). Correlation coefficients calculated using linear regression and *p*-values using the Student t test are displayed.

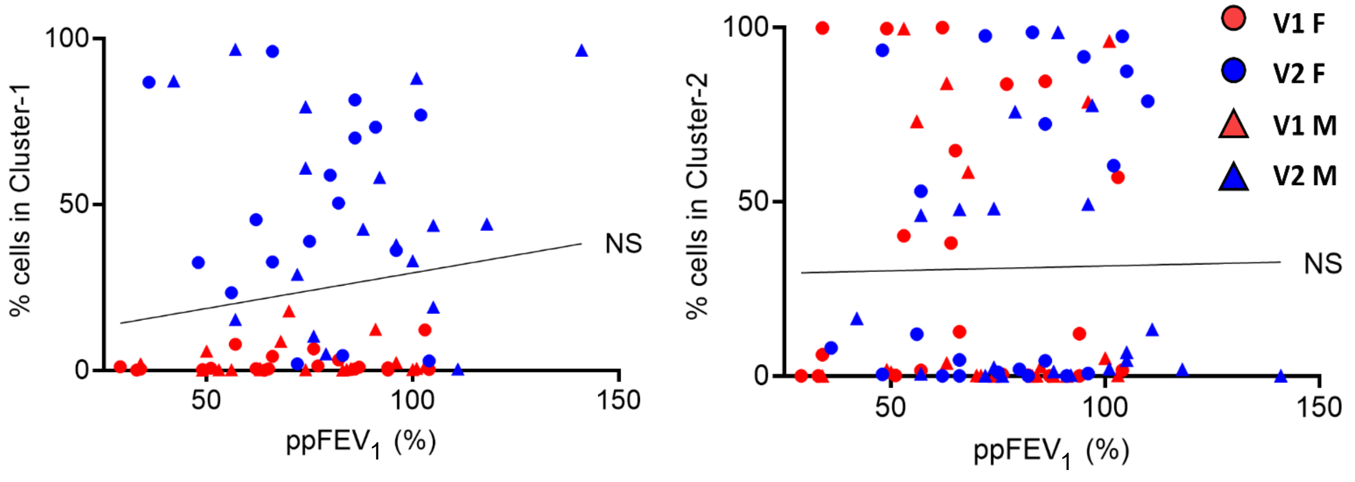

Supplementary figure S8: Effect of ppFEV$_1$ on neutrophil subsets enrichment in pwCF. Analysis of the correlation between Cluster-1 and Cluster-2 neutrophils enrichments and percent predicted forced expiratory volume in one second (ppFEV$_1$) in V1 male patients (red triangles), V2 male patients (blue triangles), V1 female patients (red circles) and V2 female patients (blue circles). Correlation coefficients were calculated using linear regression and p-values were calculated using the Student t test.
